# In Silico Design, Optimization, and Evaluation of a Multi-Epitope Vaccine Targeting the *Clostridium perfringens* Collagen Adhesin Protein

**DOI:** 10.3390/microorganisms13051147

**Published:** 2025-05-16

**Authors:** Dhiraj Chundru, Shailes Bhattrai, Madhusudan Timilsina, Hyun Lillehoj, Zhifeng Sun, Mostafa Ghanem, Charles Li

**Affiliations:** 1 Department of Veterinary Medicine, Virginia-Maryland College of Veterinary Medicine, Maryland Campus, College Park, MD 20740, USA; dhiraj16@umd.edu (D.C.); madhutim@umd.edu (M.T.); 2 Animal Biosciences and Biotechnology Laboratory, Beltsville Agricultural Research Center, Agricultural Research Service-US Department of Agriculture, Beltsville, MD 20705, USA; shailes.bhattrai@usda.gov (S.B.); hyun.lillehoj@usda.gov (H.L.); zhifeng.sun@usda.gov (Z.S.)

**Keywords:** *Clostridium perfringens*, necrotic enteritis, collagen adhesin protein (CNA), multi-epitope vaccine (MEV), CD4^+^ T cell, CD8^+^ CTL, B-cells, epitopes, immunoinformatics

## Abstract

Necrotic enteritis (NE), caused by pathogenic *Clostridium perfringens*, poses a significant threat to global poultry health, with estimated annual losses exceeding USD 6 billion. The rising incidence of NE has been associated with the reduced use of antibiotic growth promoters, underscoring the urgent need for alternative control measures such as vaccination. Collagen adhesin protein (CNA), a key virulence factor in NE pathogenesis, represents a promising vaccine target. The US Food and Drug Administration has begun phasing out animal testing requirements for biologics and monoclonal antibody drugs. In this study, a computational multi-epitope vaccine (MEV) targeting CNA was designed by integrating predicted Cluster of Differentiation (CD)4^+^ helper T lymphocyte (Th), CD8^+^ cytotoxic T lymphocyte (CTL), and B-cell epitopes. Bioinformatics tools were used to identify immunogenic, antigenic, and non-allergenic epitopes assembled into a 115-amino-acid peptide vaccine construct. The candidate demonstrated strong stability and solubility. In silico immune simulation predicted robust immune responses, including elevated IgG and IgM antibody levels, plasma cell proliferation, Th memory formation, and CTL activation, comparable to responses elicited by a full-length CNA. These findings support the potential of the designed peptide as one of the multiple effective NE vaccine components, offering a promising alternative to antibiotic-based approaches in poultry disease management.

## 1. Introduction

*Clostridium perfringens* is a common Gram-positive anaerobic bacterium responsible for various diseases in animals, including gas gangrene, food poisoning, and necrotic enteritis (NE) [1]. Among the identified strains, *C. perfringens* type-G, which produces alpha-toxin (CPA) and the NE B-like pore-forming toxin (NetB), is the primary causative agent of NE [2,3,4], which imposes an estimated annual economic burden of USD 6 billion on the global broiler industry [5]. To combat NE disease, scientists have been endeavoring to develop multiple types of vaccines [6], including inactivated toxoid vaccines [4,7], recombinant protein subunits [8,9], and live-attenuated strains targeting key virulence factors, such as CPA and NetB [10,11,12]. While toxoid-based vaccines have been used with varying success, their production can be costly and sometimes elicit suboptimal immunity [6]. Recombinant vaccines targeting NetB and other toxins have shown promise in reducing NE lesions in poultry models, yet achieving broad and lasting protection remains challenging [13]. Live-attenuated and probiotic-vectored vaccine strategies have also emerged, but concerns persist regarding safety, regulatory approval, and efficacy in the presence of pre-existing vector immunity [6]. However, most current vaccine candidates, which primarily focus on toxoids such as NetB and CPA, still require thorough validation in animal models. Additionally, other critical virulence factors involved in the early stages of NE pathogenesis should be considered as potential targets for effective intervention.

In chickens affected by NE, histological examinations reveal dense layers of *C. perfringens* cells adhering to lesions in the small intestine, highlighting the critical role of bacterial adherence in NE pathogenesis [14]. Bacterial adherence to host tissues is mediated by cell surface structures such as adhesins, flagella, and pili [15].

The collagen adhesin protein (CNA) located on the surface of *C. perfringens* is encoded by the *cna* gene and represents a critical virulence factor that enables *C. perfringens* to adhere to host extracellular matrix proteins, facilitating tissue colonization and subsequent pathogenesis [16,17]. In *netB*-positive *C. perfringens* strains, a 9 kb chromosomal locus (VR-10B), which consists of seven open reading frames, is predicted to encode an adhesive sortase-dependent pilus and a two-component system [18]. While sortase-dependent pili play a well-established role in the virulence of many Gram-positive pathogens, including *Corynebacterium diphtheriae* [19], *Enterococcus faecalis* [20], *Streptococcus pyogenes* [21], and *Streptococcus agalactiae* [22], their role in *C. perfringens* has only recently been explored [16]. CNA is a component of a sortase-assembled pilus structure, acting as the tip adhesin that specifically interacts with collagen types I through V, which are abundant in the intestinal extracellular matrix [16]. Sortase enzymes facilitate pilus assembly by covalently linking these pilin subunits and anchoring the structure to the bacterial cell surface. Strains harboring the *cnaA* gene demonstrate enhanced collagen-binding capacity with increased pathogenicity [23]. In contrast, the deletion of *cnaA* significantly reduces bacterial attachment to various collagen types (collagen types IV and V and gelatin), compared to wild-type strains [16,24]. Additionally, broiler chickens challenged with *cnaA*-null mutant *C. perfringens* strains displayed a lower incidence of NE in these studies, further underscoring the importance of CNA in bacterial adherence and pathogenicity [16,23]. These findings collectively indicate CNA being a promising target for vaccine development against *C. perfringens*-induced NE.

Immunization or vaccination enhances the host immune system’s ability to recognize and target pathogens, making them effective tools for controlling infectious diseases [25,26,27]. Advances in bioinformatics and a deeper understanding of the host-pathogen immunobiology have accelerated the development of potent epitope-based vaccines with improved efficacy and low cost [28]. Previous studies have explored the development of multi-epitope vaccines (MEVs) against *C. perfringens* using bioinformatics approaches, targeting the NetF toxin [29] and other extracellular antigenic proteins [30]. However, most vaccine research against *C. perfringens* has primarily focused on its toxigenic proteins, with relatively little attention given to structural adhesin proteins such as CNA. In a robust experimental NE model, broiler chickens immunized with CNA alone or in combination with other antigens-including CPA, NetB, fructose-1,6-bisphosphate aldolase (FBA), and zinc metalloprotease (ZM)-exhibited protection against lethal *C. perfringens* challenge [8]. Despite these promising results, the underlying molecular mechanisms of protection remain poorly understood, posing a challenge for the development of more effective vaccines.

Immunoinformatic-based multi-epitope prediction for B- and T-cell epitopes has emerged as a key approach in vaccine development during recent years. B-cell epitopes induce antibody responses that neutralize bacterial adhesion, prevent colonization and pathogenesis, and establish immune memory for long-term protection [31,32]. Meanwhile, T-cell epitopes-comprising helper T (Th) cells and cytotoxic T lymphocytes (CTLs)-play a vital role in controlling bacterial spread by releasing cytokines, eliminating infected cells, and assisting B cells in antibody class switching [33]. Although *C. perfringens* is traditionally regarded as an extracellular pathogen, emerging studies indicate that certain strains can invade toxin-damaged tissues or be internalized by epithelial cells under specific strain-dependent conditions [34]. This internalization often relies on toxin production, extracellular enzymatic activity that disrupts epithelial integrity, or binding to specific host receptors, thereby facilitating bacterial entry [34,35]. Such mechanisms may contribute to immune evasion and persistent colonization. Moreover, *C. perfringens* infection has been shown to activate CD8^+^ T cells and elicit host cellular immune responses [36,37]. Therefore, the inclusion of predicted CD8^+^ T cell epitopes in our vaccine design was intended to broaden and enhance the overall immune protection.

The US Food and Drug Administration (FDA) has announced plans to begin phasing out animal testing requirements for biologic drugs, including monoclonal antibodies, and with the possibility of expanding these changes to other drug categories in the future. This shift reflects a growing emphasis on human-relevant approaches, such as AI-based models and advanced humanoid systems. Unlike traditional vaccine development, computational methods leverage pathogen genome databases to identify potential antigenic epitopes, which can elicit a targeted immune response and enhance vaccine efficacy [38]. This study outlines a comprehensive strategy for developing, optimizing, and evaluating an MEV against CNA by utilizing CD4^+^ Th, CD8^+^ CTL, and B-cell epitopes through advanced bioinformatics approaches.

## 2. Materials and Methods

### 2.1. Raw Data Source and Sequence Retrieval

The consensus CNA RefSeq protein sequence of *C. perfringens* (WP_057230734.1) was retrieved from the National Center for Biotechnology Information (NCBI). The sequence encoded a CNA protein composed of 647 amino acids.

### 2.2. Prediction and Selection of Epitopes

**Figure 1:**
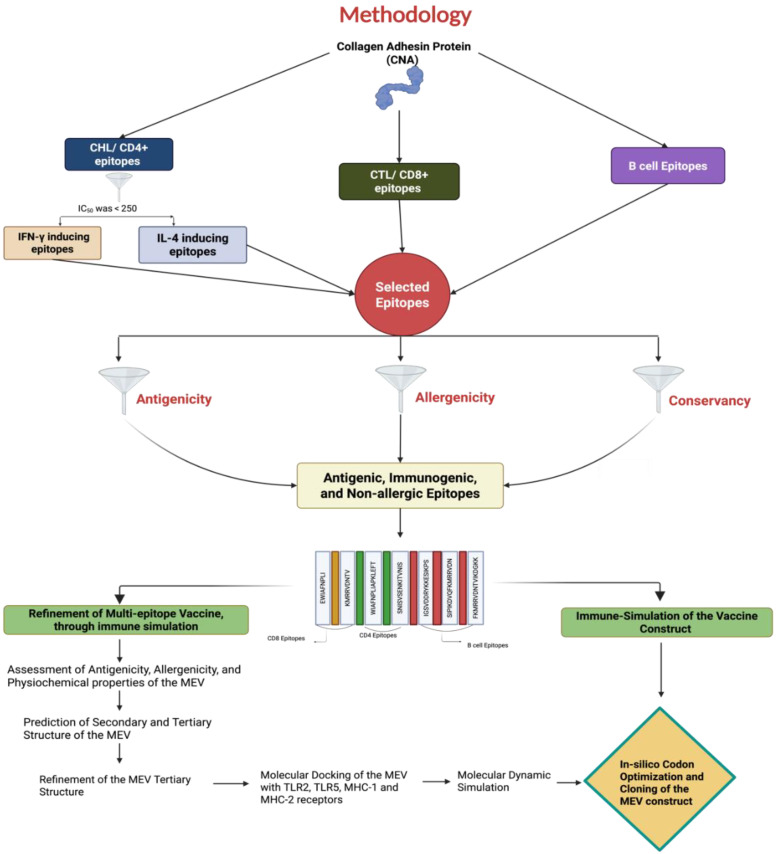
A diagram illustrating the proteomics-based pipeline employed in this study to identify T- and B-cell epitopes from the *Clostridium perfringens* collagen adhesin protein (CNA) for creating a multi-epitope vaccine (MEV) using an immunoinformatics approach.

Various bioinformatic tools were utilized to predict and select major histocompatibility complex (MHC) I, II, and B-cell epitopes. Due to the limited availability of immunoinformatic tools for chicken alleles, human leukocyte antigen (HLA) alleles were used as a substitute for predicting chicken MHC-I and MHC-II epitopes. Studies have shown that chicken BF haplotypes share similar anchor residues to mammalian MHC, particularly for 8–9 mer peptides, supporting the use of human alleles for epitope prediction in this study [39]. The detailed procedures for the study are outlined in Figure 1.

#### 2.2.1. Prediction of CD8^+^ CTL Epitopes

To initiate a protective immune response against infection, antigen presentation to CTLs via MHC-I is crucial. The Next-Generation IEDB tool for T-cell prediction-MHC Class I (accessed on 3 April 2024 from https://nextgen-tools.iedb.org/pipeline?tool=tc1) to predict nonamers (9-mers) capable of binding to MHC-I HLA alleles and eliciting a CD8^+^ CTL response [40]. This tool had an inbuilt immunogenicity feature that assessed the immunogenic potential of the predicted CD8^+^ CTL epitopes. The MHC-I epitopes were predicted using NetMHCpan BA 4.1 [41], with selection criteria set to a peptide length of nine amino acids, an inhibitory concentration 50 (IC_50_) value < 250, and immunogenicity scores > 0.

#### 2.2.2. Prediction of CD4^+^ Th Epitopes

Stimulation of Th cells by MHC-II epitopes derived from the CNA protein may induce both cellular and humoral responses, facilitating antibody maturation and class switching. Th epitopes were predicted using the IEDB MHC-II binding server (accessed on 3 April 2024 from http://tools.iedb.org/mhcii/) with the stabilization matrix alignment (SMM-align) method, targeting peptides with 15 amino acids and an IC_50_ threshold below 250. The identified epitopes were further analyzed using the IFNepitope (accessed on 3 April 2024 from http://crdd.osdd.net/raghava/ifnepitope/) and IL4pred (accessed on 3 April 2024 from http://crdd.osdd.net/raghava/il4pred/) tools to assess their potential to induce cytokines interferon-gamma (IFN-γ) and interleukin-4 (IL-4), respectively. Epitopes predicted to induce both IFN-γ and IL-4 cytokines were selected for further evaluation.

#### 2.2.3. Prediction of B-Cell Epitopes

B-cell epitopes play a crucial role in stimulating antibody production and promoting humoral immunity. To identify antigenic epitopes capable of inducing a B-cell response, the ABCpred server (accessed on 3 April 2024 from http://osddlinux.osdd.net/raghava/abcpred/) was utilized [42]. The prediction parameters were set with a window length of 12–16 amino acids and a threshold value of 0.51.

#### 2.2.4. Antigenicity, Conservancy, and Allergenicity of the Selected Epitopes

An ideal vaccine must induce a safe and effective immune response in the host. The antigenicity of the vaccine construct was predicted using Vaxijen v2.0 (accessed on 3 April 2024 from http://www.ddg-pharmfac.net/vaxijen/VaxiJen/VaxiJen.html) with a threshold of 0.5. Epitope conservancy was analyzed using the IEDB web tool (accessed on 3 April 2024 from http://tools.iedb.org/conservancy/), while allergenicity was assessed using the AllerTop v2.0 prediction tool [43,44]. Only sequences classified as antigenic, conserved, and non-allergenic were selected for MEV construction.

### 2.3. Merging of Epitopes and Construction of the MEV

**Figure 2:**
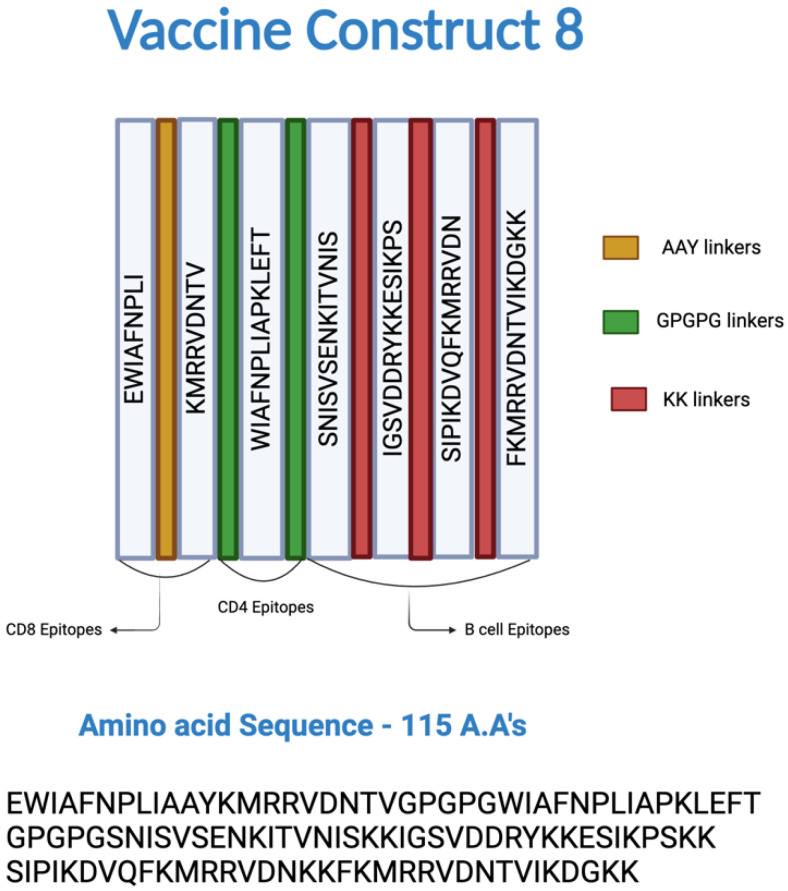
Epitopes and linkers involved in the construction of multi-epitope vaccine (MEV) Construct 8. The CD8^+^, CD4^+^, and B-cell epitopes were joined by AAY, GPGPG, and KK linkers, respectively.

After identifying the most suitable epitopes for optimal CD8^+^, CD4^+^, and B-cell responses and screening them for antigenicity, allergenicity, and conservancy using various immunoinformatic tools, the selected epitopes were fused together with specific linkers to prevent the formation of junctional epitopes [45]. To design an MEV construct, selected CTL-, Th-, and B-cell epitopes that may elicit cellular and humoral responses were linked using AAY, GPGPG, and KK linkers, respectively [46]. The addition of linkers enhanced the flexibility and stability of the peptide constructs. An initial construct was created by assembling all the selected epitopes (Figure 2) and assessed using in silico immune simulation data from the C-IMMSIM tool (accessed on 3 April 2024 from https://kraken.iac.rm.cnr.it/C-IMMSIM/index.php). For immune simulation, all parameters were set to default. Two injections were simulated to be administered on days 1 and 14, with a two-week interval. The immune responses of this initial vaccine construct (Supplementary File S1) were compared to those of the CNA protein construct (Supplementary File S2), evaluating parameters such as peripheral blood lymphocyte (PBL) cells, B memory cells, active Th cells, active CTL cells, and total immunoglobulins. The goal was to determine if the construct was comparable to or better than the whole CNA. Several vaccine constructs were generated and assessed to produce the smallest yet most effective version. Due to the impracticality of theoretically constructing and evaluating all possible combinations, a systematic approach of deleting or adding epitopes from the initial vaccine construct was used to optimize it.

#### 2.3.1. Assessment of Antigenicity, Allergenicity, and Physiochemical Properties of the MEV

The final MEV construct was screened for sequence homology with the host (*Gallus gallus*) protein using a BLASTp search, using NCBI blastp suite (BLAST+ 2.14.0: 25 April 2023) accessed on 3 May 2024. The antigenicity of the final MEV construct was evaluated with the VaxiJen v2.0 server (accessed on 3 May 2024 from https://www.ddg-pharmfac.net/vaxijen/VaxiJen/VaxiJen.html), and allergenicity was predicted using the Allertop v2.0 server (accessed on 3 May 2024 from https://www.ddg-pharmfac.net/allertop_test/). The physiochemical properties of the MEV construct, including molecular weight, isoelectric point, instability, thermostability, and other attributes, were assessed using the Expasy ProtParam server (accessed on 3 May 2024 from https://web.expasy.org/protparam/) [42]. The solubility of the MEV was predicted using the Protein-Sol server (accessed on 3 May 2024 from https://protein-sol.manchester.ac.uk/) [43].

#### 2.3.2. Prediction of Secondary and Tertiary Structures of the MEV

The secondary structures of the MEV, including α-helices, β-sheets, β-turns, random coils, and extended chains, were predicted using the PSIPRED workbench (accessed on 3 May 2024 from https://bio.tools/psipred) [47,48]. The interaction of the MEV polypeptide with the various proteins, including immune system receptors, was influenced by the protein conformation. The tertiary structure of the MEV construct was predicted using the ColabFold platform (accessed on 3 May 2024 from https://colab.research.google.com/github/sokrypton/ColabFold/), which enabled accelerated protein structure prediction through the AlphaFold2 machine learning model [49,50].

#### 2.3.3. Refinement of the MEV Tertiary Structure

After the initial prediction of the MEV tertiary structure, potential inaccuracies may arise due to limitations in predictive algorithms or the absence of an appropriate template. To enhance the structural model, molecular refinement was applied, focusing on optimizing side-chain conformations, minimizing steric clashes, and improving overall stereochemical quality. The top-ranked relaxed models of the MEV, predicted by AlphaFold2, were further refined using the Refine2 tool of the GalaxyRefine server (accessed on 3 May 2024 from https://galaxy.seoklab.org/cgibin/submit.cgi?type=REFINE) [51,52]. The server generated ten refined models, which were evaluated based on various metrics, including root mean square deviation (RMSD), MolProbity score, steric clashes, percentage of poor rotamers, percentage of amino acid residues in the Ramachandran-favored region, and energy score. These metrics collectively assessed the structural accuracy, stability, and overall quality of the protein models. The top-ranked refined models were further analyzed and validated using the PROCHECK tool available in the SAVES v6.0 server (accessed on 3 May 2024 from https://saves.mbi.ucla.edu/) [53]. Backbone dihedral angles, side-chain conformations, residue properties, bond lengths and angles, G-factors, and planar group deviations of the predicted refined models were analyzed to assess their structural quality. The two highest-ranked refined tertiary structure models of the MEV construct were selected for subsequent molecular docking evaluation with various immune receptors.

### 2.4. Molecular Docking of the MEV with Toll-like Receptor (TLR) 2, TLR5, MHC-I, and MHC-II Receptors

The three-dimensional protein structures of chicken TLR2, TLR5, MHC-I, and MHC-II receptors were downloaded from UniProt in protein database (PDB) format (UniProtKB entries: C4PBR8-1, C4PCL5, O46790, and Q4U5Z6-1 + B5BSA0-1). Molecular docking analysis (MDA) was performed using the ClusPro v2.0 server (accessed on 3 May 2024 from https://cluspro.bu.edu/login.php?redir=/home.php) to analyze the interaction of the MEV with these receptors [54,55,56]. The interaction between the MEV and the receptors was predicted by simulating rigid docking of the proteins across numerous possible conformations. The predicted MEV-receptor complexes were ranked based on cluster size and energy states, with the complex having the largest cluster size and lowest energy state considered to have the highest probability of biologically relevant interaction. The high-ranked MEV-receptor docked complex was visualized using UCSF ChimeraX v1.8 software [57].

### 2.5. Molecular Dynamic Simulation (MDS)

The highest-ranked MEV-receptor complexes were subjected to molecular dynamics simulation (MDS) using the iMODS server (accessed on 3 May 2024 from http://imods.chaconlab.org) with default parameters. The iMODS MDS tool performed normal mode analysis (NMA) to evaluate the intrinsic flexibility and potential conformational changes in the MEV-receptor complex. This analysis predicted deformability, mobility, eigenvalues, and atomic displacements, providing visual representations of these dynamic behaviors [58]. The stability of the protein was assessed through the deformability plot of the polypeptide chain, B-factor values, eigenvalue, covariance matrix, and elastic network.

### 2.6. Immune Simulation of the MEV and Native CNA Protein

The MEV construct was subjected to in silico immune simulation using tools available on the C-ImmSim server (accessed on 3 May 2024 from https://kraken.iac.rm.cnr.it/C-IMMSIM/index.php) with default parameters. The assessment was based on key immune response indicators, including the number of elicited PLB cells, B memory cells, active Th cells, active CTL cells, and total immunoglobulins, as provided in the C-ImmSim results. This evaluation aimed to determine whether the MEV construct was comparable to, superior to, or inferior to the native CNA protein. Additionally, a comparative analysis of the final MEV construct was conducted against multiple in silico MEV constructs synthesized with different combinations of B-cell and T-cell epitopes, following the same methodology.

### 2.7. In Silico Codon Optimization and Cloning of the MEV Construct

The in silico codon optimization of the MEV construct for expression in *Escherichia coli* was performed using GenScript’s GenSmart™ Codon Optimization tool (accessed on 3 May 2024 from https://www.genscript.com/gensmart-free-gene-codon-optimization.html). This tool utilizes advanced proprietary algorithms to optimize codon usage specifically for high-level expression in *E. coli*. Throughout the optimization process, the GC content and sequence length of the MEV construct were fine-tuned to maximize expression efficiency [59]. Following optimization, the MEV sequence was inserted into the pET28a (+) plasmid vector between the EcoRI (GAATTC) and XhoI (CTCGAG) restriction sites. The plasmid containing the MEV construct was then visualized using the Benchling online tool (accessed on 3 May 2024 from https://benchling.com/) to confirm the successful insertion and overall integrity of the plasmid.

## 3. Results

### 3.1. Prediction of T-Cell and B-Cell Epitopes

#### 3.1.1. Cytotoxic T-Cell/CD8^+^ T-Cell Epitope Prediction

The Next-Generation IEDB Tool for T-cell prediction-MHC Class I produced 640 unique epitopes. Further selection of epitopes using the parameter of IC_50_ < 250 resulted in 95 unique epitopes. Out of these 640 epitopes, only 58 epitopes were found to be potentially immunogenic by the Class I pMHC immunogenicity module of the Next-Generation IEDB Tool used.

#### 3.1.2. Helper T-Cell/CD4^+^ T-Cell Epitope Prediction

The IEDB MHC-II binding tool identified a total of 633 Th epitopes. Among these, 480 were predicted as IL-4-inducing epitopes using the IL4pred tool. Additionally, the IFN epitope tool identified 218 epitopes capable of producing IFN-γ. Of these, 172 epitopes were predicted to induce both IL-4 and IFN-γ and were selected for further analysis.

#### 3.1.3. B-Cell Epitope Prediction

The ABCpred server predicted a total of 68 unique B-cell epitopes, all of which were selected for further analysis.

#### 3.1.4. Antigenicity, Conservancy, and Allergenicity of Selected Epitopes

Among the 58 identified CTL epitopes, 27 were predicted to be antigenic, but only 2 were non-allergenic. Of the 172 selected Th epitopes, 94 were predicted to be antigenic, with 12 classified as non-allergenic. Similarly, among the 68 predicted B-cell epitopes, 45 were antigenic, and 8 were non-allergenic. For the initial MEV construct, the final selection included two CTL epitopes, along with the top four non-allergenic Th and B-cell epitopes.

### 3.2. Construction, Evaluation, and Refinement of the MEV

All selected epitopes were joined using specific linkers: AAY, GPGPG, and KK linkers for the CD8^+^ T-cell, CD4^+^ T-cell, and B-cell epitopes, respectively, to prevent the formation of junctional epitopes, enhance peptide flexibility, and improve the overall stability of the construct. The amino acid sequence of the epitopes used in the construction of the MEV are listed in Table 1 and Figure 2, and the amino acid sequence of the MEV construct was “EWIAFNPLIAAYKMRRVDNTVGPGPGWIAFNPLIAPKLEFTGPGPGSN ISVSENKITVNISKKIGSVDDRYKKESIKPSKKSIPIKDVQFKMRRVDNKKFKMRRVDNT VIKDGKK”. Among the limited combinations, MEV Construct 8 was identified as the smallest and most comparable, surpassing the CNA protein in certain immune parameters.

### 3.3. Assessment of Antigenicity, Allergenicity, and Physiochemical Properties of the MEV

The BLASTp analysis showed that the MEV was not homologous with the host protein (*Gallus gallus domesticus*) (Table S1). The MEV construct was found to be non-allergenic based on the AllerTOP v2.0 evaluation. Similarly, the MEV candidate was found to be strongly antigenic, with an overall score of 1.0494, where the threshold for antigenicity was 0.4 (Figures S1.1 and S1.2). The physiochemical properties of the MEV are presented in Table 2. The Expasy ProtoParam (accessed on 3 May 2024 from https://web.expasy.org/protparam/) server predicted the molecular weight of the MEV protein to be 12.98 kDa, with a theoretical isoelectric point score of 10.26, indicating that the protein was slightly basic. The MEV had an instability index of 30.28, suggesting a relatively stable protein, and an aliphatic index of 77.91, indicating very high thermal stability. The grand average of the hydropathicity (GRAVY) index was −0.617; the negative sign suggested that the protein was highly hydrophobic, which may facilitate its uptake into cellular compartments through the cell membrane. The average half-life of the MEV in mammalian reticulocytes in vitro was estimated to be around 1 h, and it was estimated to be more than 10 h in *E. coli* in vivo. The solubility of the protein, predicted by Protein-Sol, was 0.848, indicating that the protein was highly soluble upon expression.

### 3.4. Secondary and Tertiary Structures of the MEV

**Figure 3:**
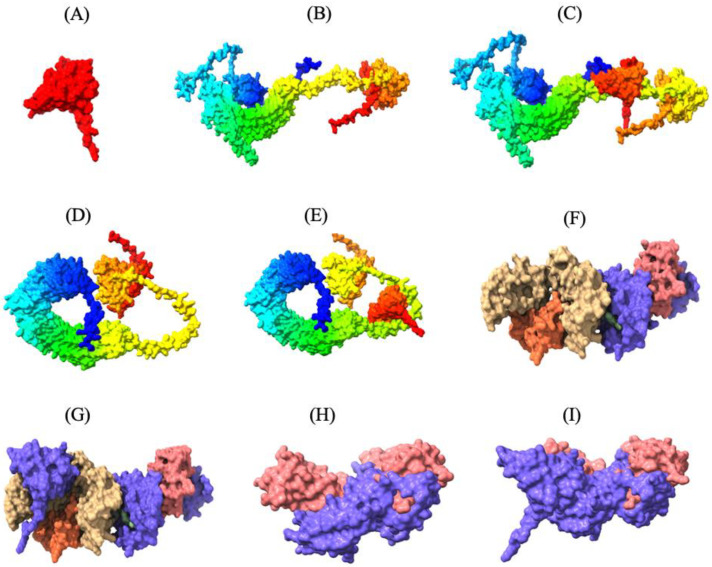
Three-dimensional tertiary structure of the multi-epitope vaccine (MEV) Construct 8 with immune receptors in chickens using Chimera-X: three-dimensional (**A**) surface representation of the MEV construct shown in red to highlight its structural conformation, (**B**,**D**,**F**,**H**) surface representations of the undocked chicken immune receptors; TLR2, TLR5, MHC-I, and MHC-II, respectively; (**C**,**E**,**G**,**I**) surface representations of the MEV--receptor docked complexes showing binding interfaces for MEV with TLR2, TLR5, MHC-I, and MHC-II, respectively.

The secondary structure of the MEV, as predicted by PSIPRED workbench, was composed of 13.04% α-helix, 30.43% β-strands, and 56.52% random coils (Figure S2). The AlphaFold2 machine learning model on the ColabFold platform yielded five unrelaxed and five relaxed tertiary structures of the MEV. The unrelaxed structure represented the initial raw prediction based solely on the neural network’s interpretation of the protein sequence, whereas the relaxed protein structures underwent an energy minimization step and considered physical forces and steric clashes, resulting in structures that were generally more realistic [49]. Compared to the unrelaxed tertiary models of the MEV, the relaxed models were validated to be in a lower energy state and exhibited a higher proportion of amino acid residues in the favorable quadrant of the Ramachandran plot (Figure 3). Thus, the two top-ranked relaxed models of MEV were selected for subsequent downstream molecular refinement.

### 3.5. Molecular Refinement of the Tertiary Structure of the MEV

Based on the evaluation of the Ramachandran plot scores, the two highest-ranked relaxed AlphaFold2 predicted models were subjected to molecular refinement using the GalaxyRefine server. The GalaxyRefine server provided ten refined protein models for each of the AlphaFold2 models. Model-2, derived from the AlphaFold2 Rank-2, was selected for downstream processing due to its lower MolProbity score, Clash score, and GALAXY energy score, compared to the other models (Table S2). Although Model-1 had a lower energy state, Model-2 had a higher proportion of amino acid residues located in the favored regions of the Ramachandran plot, a lower MolProbity score, and was slightly more deviated from the input structure compared to Model-1. According to PROCHECK evaluation, the refined model exhibited better structural quality, with 85.4% of amino acid residues in core regions in the Ramachandran plot, improved dihedral and overall G-factors (−0.09 and −0.10, respectively), and fewer labeled residues compared to the unrefined model, which had only 75.0% of amino acid residues in core regions and poorer dihedral and overall G-factors (−0.58 and −0.27, respectively) (Table 3). The table contrasts key parameters obtained from PROCHECK evaluations, including Ramachandran plot statistics, side-chain parameters, bond length/angle deviations, and G-factors. The refined model exhibited superior performance in core region residues, lower deviations, and better overall G-factors, indicating a more reliable protein conformation. This validated that the refined tertiary structure of the MEV was closer to the natural conformation of the protein.

### 3.6. Molecular Docking of the MEV with TLR2, TLR5, MHC-I, and MHC-II Receptors

Thirty MEV-receptor complex models were predicted by ClusPro v2.0 using rigid-body docking and clustering of similar conformations. The docked models were then scored and ranked based on cluster size and interaction energy. Docked models with the largest number of clusters in ClusPro are more likely to represent the natural interaction between the MEV and receptors, as a higher number of docking solutions converging on a similar binding mode suggests a more reliable and consistent prediction of the natural interaction. The MEV-MHC-II docking model, with a cluster size of 115 and a minimum energy score of −794.2 kcal/mol, was predicted to resemble the natural interaction most closely. Similarly, the optimal MEV-MHC-I docking model had 72 clusters and a minimum energy score of −790.2 kcal/mol. Furthermore, the optimal docking model of MEV with TLR2 displayed 157 clusters with a lowest energy score of −1046.4 kcal/mol, while the best MEV-TLR5 docking model revealed 84 clusters with a lowest energy score of −998.1 kcal/mol (Table S3.1–3.4). These MEV–receptor docking models, which are most likely the closest to the natural binding interaction, were subsequently visualized using UCSF ChimeraX v1.8, highlighting the strong binding interaction between the MEV construct and the immune receptors (Figure 3).

### 3.7. Molecular Dynamic Simulation

**Figure 4:**
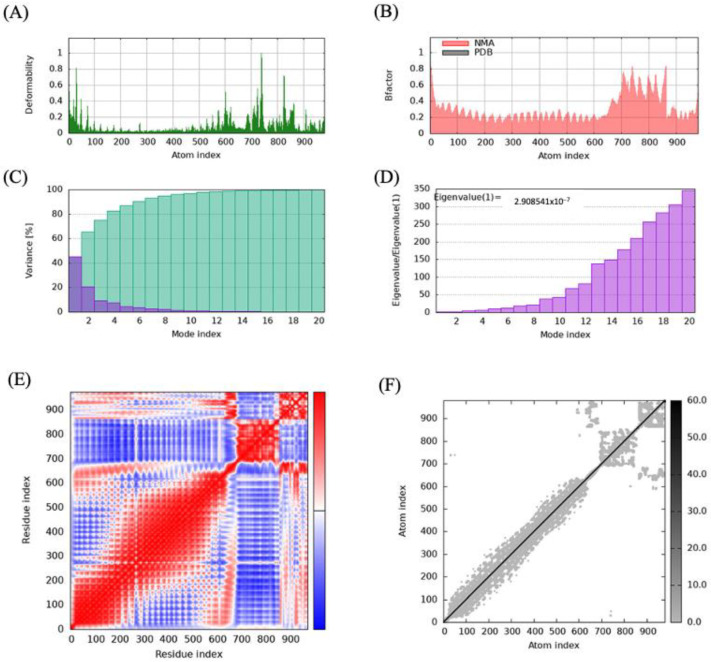
Molecular dynamics simulation analysis of the multi-epitope vaccine (MEV) Construct 8−TLR5 complex: (**A**) deformability analysis of the complex, depicting regions of high flexibility; (**B**) B-factor map showing fluctuations in atomic positions during the simulation; (**C**) variance analysis demonstrating the contribution of different modes to the overall motion; (**D**) eigenvalue plot illustrating the stiffness and stability of the system based on the principal modes of motion; (**E**) covariance matrix illustrating correlated, anti-correlated, and irrelevant motions within the complex in red, blue, and white colors, respectively; and (**F**) elastic network model representing the connections and constraints among residues.

Protein mobility and stabilization were predicted using normal mode analysis (NMA) on the iMODS server, which relies on the coordinates of the MEV-receptor complex. The analysis revealed that all the vaccine–receptor complexes had deformable regions interspersed among relatively rigid regions, as shown in the deformability maps (Figure 4 and Figure S3.1–3.3). The B-factor value, which quantifies the vibrational motion of atoms, showed higher values in flexible regions that may play roles in protein-protein interactions. The B-factor values were largely similar to those from the deformability map, which indicates the robustness of the protein structure prediction. Based on the analysis of the deformability map and B-factor values, the MEV construct largely exhibited compatibility with the natural dynamics of the receptors, with no major conformational distortion observed in the MEV-receptor complex, as the deformations were localized near the binding interface (Figure 3D,E and Figure 4A).

Similarly, the analysis of the different vibrational modes of the protein complex revealed that few modes with low eigenvalues were critical in driving the protein’s global movement and the dynamic behavior of the complexes (Figure 4D).

### 3.8. Immune Simulation of the MEV and Native CNA Protein

Simulation of the immune response to MEV administration was evaluated using the C-IMMSIM immune simulation server. The simulation showed that MEV Construct 8 was more immunogenic compared to the native CNA protein, despite being a smaller protein (Table 4). MEV Construct 8 significantly outperformed the native CNA protein in eliciting a comprehensive immune response, with notably higher total IgG + IgM levels (~90,000) compared to that of native CNA (~75,000).

The IgG1 response was also significantly higher in MEV Construct 8 (~100 vs. 50 in native CNA), reflecting a better serum immune response, class-switching, and long-term immunity potential. Additionally, MEV Construct 8 demonstrated a stronger ability to sustain a robust Th memory cell population (~1800 vs. 1200 in native CNA), indicating enhanced memory cell formation. Overall, MEV Construct 8, designed through an immunoinformatics-driven approach, exhibited a highly potent and durable immune response, making it a more effective vaccine candidate than the native CNA protein. While multiple vaccine constructs with different combinations of T- and B-cell epitopes were assessed, MEV Construct 8 outperformed all others, solidifying its potential as the optimal candidate.

**Figure 5:**
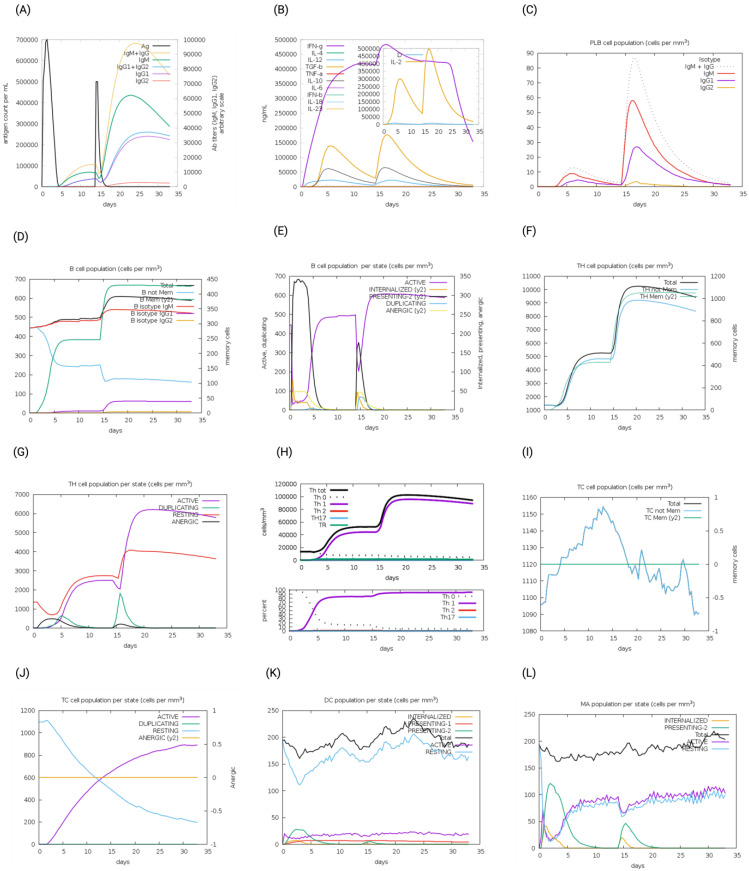
In silico immune system simulations produced by the C-ImmSim server for the collagen adhesin protein (CNA): (**A**) levels of antibodies after primary and secondary immune response, (**B**) cytokine and interleukins production, (**C**) PLB-cell population per isotype, (**D**) B-cell population, (**E**) B-cell population per state, (**F**) T helper (Th)-cell population, (**G**) Th-cell population per state, (**H**) polarization of T-cell response to Th1, (**I**) cytotoxic T-cell population, (**J**) cytotoxic T-cell population per state, (**K**) dendritic cell population per state, and (**L**) macrophage population per state.

**Figure 6:**
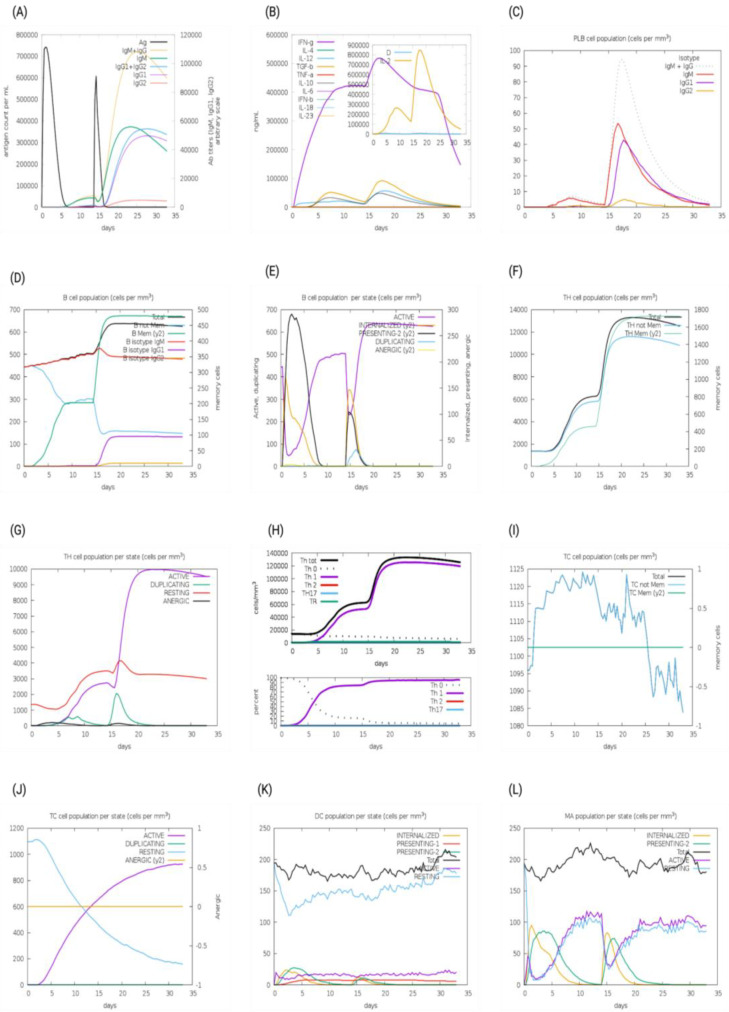
In silico immune system simulations produced by the C-ImmSim server for multi-epitope vaccine (MEV) Construct 8: (**A**) levels of antibodies after primary and secondary immune response, (**B**) cytokine and interleukins production, (**C**) PLB-cell population per isotype, (**D**) B-cell population, (**E**) B-cell population per state, (**F**) T helper (Th)-cell population, (**G**) Th-cell population per state, (**H**) polarization of T-cell response to Th1, (**I**) cytotoxic T-cell population, (**J**) cytotoxic T-cell population per state, (**K**) dendritic cell population per state, and (**L**) macrophage population per state.

The immunogenic profiles of the whole CNA protein vaccine and MEV Construct 8 are also shown in Figure 5 and Figure 6, respectively. After immune simulation, antibody levels (IgM  +  IgG, IgM, IgG1  +  IgG2, IgG1, and IgG2) significantly increased in both vaccine types (Figure 5A and Figure 6A), with MEV Construct 8 showing even higher levels (Figure 6A). Similar increases in B- and T-cell (both Th and CTL) populations were observed with both the CNA vaccine (Figure 5C–J) and Construct 8 (Figure 6C–J), with Construct 8 demonstrating stronger stimulatory effects. As observed, an increase in population of Th cells accompanied by an increase in the Th1 population suggested a polarization of the T-cell response that could help the humoral response. Additionally, the rise in dendritic cells and macrophages in both the CNA vaccine (Figure 5K,L) and Construct 8 (Figure 6K,L) indicated efficient antigen processing and delivery to CD4 ^+^ and CD8 ^+^ cells, making the subunit vaccine as effective as the full-length CNA vaccine. Furthermore, MEV Construct 8 produced higher levels of cytokines, including IFN-γ, IL-2, and IL-10 (Figure 6B), compared to the native CNA protein (Figure 5B), suggesting a favorable immune response from the newly designed subunit vaccine. Moreover, the Simpson index (D), a measure of diversity, increased over time in both the vaccine groups (Figure 5B and Figure 6B).

### 3.9. In Silico Codon Optimization and Cloning of the MEV Construct

**Figure 7:**
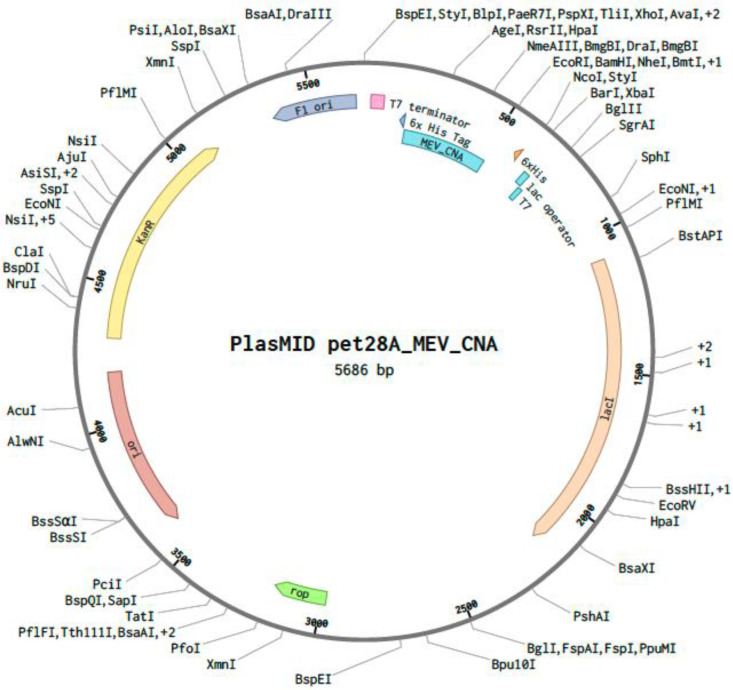
The pET28a (+) vector displaying the codon-optimized nucleotide sequence of the multi-epitope vaccine (MEV) construct for *Escherichia coli* expression. The MEV cloned sequence is represented in blue inside the vector backbone (dark gray). The cloned MEV sequence is flanked by XhoI (position 158) and EcoRI (position 509) restriction enzyme sites.

Using GenScript’s GenSmart™ Codon Optimization tool, the MEV construct underwent in silico codon optimization for cloning and expression in an *E. coli* expression vector. The optimized sequence had a final GC content of 46.38%, and the nucleotide sequence was optimized to a final length of 345 base pairs. Recognition sites of the restriction enzymes XhoI [CTCGAG] and EcoRI [GAATTC] were excluded while optimizing the MEV sequence. A custom plasmid was designed in silico by inserting the optimized MEV sequence between the XhoI and EcoRI restriction sites of the pET28a (+) plasmid on the Benchling online tool (Figure 7).

## 4. Discussion

The global poultry industry is facing economic challenges due to the increasing prevalence of NE caused by *C. perfringens* [5]. Historically, NE was controlled efficiently through prophylactic antibiotic use [60]. However, concerns over the development and spread of antimicrobial-resistant bacteria have led to a ban on the use of antibiotics [61]. Consequently, developing alternative strategies, such as vaccines, to control NE has become essential to ensure safe and cost-effective poultry production. Although previous studies focused on toxigenic proteins responsible for NE in poultry, recent research has highlighted the roles of other virulence factors [2,14,18]. These factors may aid in pathogen adherence and colonization [62], including a collagen-binding NE pilus found in *C. perfringens* strains affecting birds [16,18,24]. Thus, current strategies should also focus on developing effective vaccines that target novel proteins involved in bacterial pathogenesis.

Researchers have long aimed to enhance cost efficiency and develop effective vaccines for infectious diseases. Advances in bioinformatics, particularly immunoinformatics, have significantly accelerated the design and optimization of MEVs, enabling more precise and efficient vaccine development [28,63]. Unlike traditional vaccines, MEVs exclude non-essential components that may trigger abnormal immune responses or adverse effects [64]. In this study, the *C. perfringens* CNA protein was analyzed using immunoinformatic approaches to identify suitable T- and B-cell epitopes for MEV development. Various databases and online servers were utilized to select optimal vaccine candidates and predict immune responses induced by CTL, Th lymphocytes, and B-cell epitopes, since these cells are involved in activating humoral and cell-mediated immunity. CTLs control infection by eliminating infected cells and producing antiviral cytokines, and B cells generate antibodies [65]. Given their essential role in eliciting immune responses, the final MEVs were designed to target both T- and B-cell epitopes. Epitope selection was based on immunogenicity, allergenicity, and conservancy, ensuring that only non-allergenic, immunogenic, and conserved sequences were included in the vaccine construct. This approach aimed to generate a strong immune response while minimizing potential adverse effects [27].

MEV Construct 8, identified in this study, consisted of 115 amino acids and had a molecular weight of 12.98 kDa, aligning with previous vaccine design studies and making it well-suited for chimeric vaccine development [30,66,67]. Proteins with molecular weights below 110 kDa are generally easier to extract and more suitable for vaccine formulation [68,69]. Consistent with earlier findings [20,53,54], MEV Construct 8 exhibited favorable properties, including basicity, high stability, solubility, and the presence of aliphatic side chains. These characteristics suggest that the final vaccine construct contained high-affinity antigenic epitopes while maintaining structural stability, solubility, and thermostability. As a result, MEV Construct 8 represents a strong vaccine candidate capable of eliciting a robust immune response while minimizing potential side effects.

The secondary structure of MEV Construct 8 was predominantly composed of random coils (56.52%), which matched or exceeded the values reported in previous studies [30,66,67,70,71]. This structural feature could increase the protein’s flexibility, facilitating multiple docking interactions [72]. Additionally, similar to previous findings [30,66,67,70,71], the 3D structure of the construct showed that 85.40% of residues were in the most favored regions of the Ramachandran plot, indicating that the protein can attain a stable conformation after in vivo ribosomal synthesis [73]. Protein-protein docking and molecular dynamics simulations of MEV Construct 8 with TLR2, TLR5, MHC-II, and MHC-II demonstrated the vaccine’s ability to bind to these receptors, suggesting its potential to elicit both innate and adaptive immune responses.

MEV Construct 8 in this study effectively induced several immunogenic parameters, matching or surpassing those of the whole CNA protein. Consistent with previous findings [27,30,66,70,74,75], there were increases in B- and T-cell populations and immunoglobulins, as well as their isotypes, and cytokine levels were observed following immune stimulation in the simulation, with these levels being sustained. The increased B-cell population likely contributed to the higher production of immunoglobulins such as IgG and IgM, leading to reduced antigen levels, as observed in this study. Additionally, the Th-cell populations were higher in MEV Construct 8, compared to those in the whole CNA vaccine. These cells can secrete cytokines like IFN-γ, IL-2, and IL-10, which help mitigate pro-inflammatory responses and reduce tissue damage [33]. The positive correlation between Th-cell populations and cytokine levels of IFN-γ and IL-2, which was observed in a previous MEV study [74], aligns with our current findings, suggesting an effective cellular response by the vaccine against antigens, potentially minimizing pathogen damage. Consistent with the same study finding, an increase over time in the Simpson index (D), a measure of diversity, could suggest the development of different epitope-specific dominant clones of T cells, indicating the necessity of a diverse host immune response to NE.

Dendritic cells and macrophages, both key members of the mononuclear phagocyte system, play crucial roles in immune functions [76]. Dendritic cells are vital for antigen processing, presenting antigens to naïve T cells, and inducing T-cell tolerance and immunity, while macrophages are effective at capturing and killing microbes, scavenging apoptotic and dead cells, presenting antigens, and producing regulatory cytokines. In this study, populations of both dendritic cells and macrophages increased following immune stimulation with both the CNA vaccine and MEV Construct 8. The higher cytokine levels observed with MEV Construct 8, compared to the CNA vaccine, might be attributed to the greater efficiency in antigen processing and activation in the MEV molecule. Therefore, the elevated levels of macrophages, dendritic cells, and cytokines suggest that MEV Construct 8 may create an antibacterial environment to benefit antigen processing and immune activation. Additionally, the increase in cytokines, including IFN-γ, IL-2, and IL-10, with the MEV vaccine was also noted in previous studies [27,30]. Using computational tools for epitope prediction and vaccine design accelerates vaccine development, enabling the creation of more effective and optimized vaccines.

To enhance scalability and expression efficiency, we designed the CNA-based MEV by selecting only the most immunodominant and antigenic epitopes, thereby reducing the overall gene length, compared to the full-length CNA protein (647 aa). This design facilitates its incorporation into probiotic or bacterial vectors, which often have size constraints for stable expression. However, large-scale production of synthetic peptide vaccines still faces several practical challenges. These include manufacturing complexity related to multi-step peptide synthesis and purification, the need for cold-chain logistics to maintain peptide stability during storage and transportation, and the requirement for appropriate adjuvant formulations to enhance immunogenicity in poultry. Moreover, oral delivery systems must protect the antigen from degradation in the gastrointestinal tract, further complicating formulation. To overcome these limitations, recent advancements in recombinant expression systems-such as yeast, *Lactobacillus casei*, *Bacillus subtilis*, and plant-based platforms-offer promising alternatives. These systems can produce epitope-based vaccines at a low cost, in scalable quantities, and in forms that are more compatible with oral administration, thus improving the feasibility of deploying such vaccines in commercial poultry operations.

Mucosal immunity plays a critical role in protecting against necrotic enteritis, as *C. perfringens* primarily colonizes and damages the intestinal epithelium. Therefore, effective vaccine-induced protection should involve the robust stimulation of the gut-associated lymphoid tissue (GALT), leading to localized production of secretory IgA, activation of mucosal T cells, and recruitment of innate immune components. To enhance intestinal immune responses, targeted delivery strategies such as oral administration using probiotic-based vectors (e.g., *Lactobacillus casei* or *Bacillus subtilis*) offer promising advantages. These vectors not only survive passage through the gastrointestinal tract but can also interact directly with intestinal immune cells, facilitating antigen uptake and presentation. Additionally, such delivery systems are scalable, cost-effective, and compatible with mass vaccination strategies in poultry, making them ideal candidates for commercial application.

While computational immune simulations offer valuable insights into the immunogenic potential of vaccine constructs, it should be mentioned that they are inherently limited in their ability to fully recapitulate the complexity of biological systems. Tools like C-ImmSim simulate immune responses using predefined algorithms based on human immunological data and thus may not accurately reflect avian-specific immune dynamics. In our simulation, the MEV construct could induce a strong primary immune response characterized by elevated levels of IgM and a robust secondary response marked by increased IgG and memory B cells. Additionally, C-ImmSim predicted the induction of cytokines such as IFN-γ and IL-2, indicating the activation of Th1-type cell-mediated immunity, which is essential for enhancing phagocytic clearance. However, the actual in vivo immunogenicity and efficacy of the vaccine may be influenced by several factors, including host-specific antigen-processing pathways, immunodominance hierarchies that prioritize certain epitopes over others, and variability in epitope presentation across MHC haplotypes in diverse poultry lines. These variables may result in differential immune activation, incomplete protection, or unintended immune tolerance. Thus, while in silico predictions provide a rational basis for vaccine design, experimental validation in target species remains essential to confirm immune responses, safety (local and systemic adverse effects), and protective efficacy under real-world poultry farming conditions. It is recommended that emerging chicken-specific epitope prediction tools be integrated in the future, once they become available.

In future animal experiments, beyond measuring absolute body weight gain and relative growth rate, the immune responses to the MEV in vaccinated chickens could be evaluated using a range of comprehensive parameters. These include: measuring serum antibody titers (immunoglobulin Y, IgY) and mucosal antibody titers (immunoglobulin A, IgA) against the selected epitopes by enzyme-linked immunosorbent assay; analyzing cytokine profiles (such as IFN-γ, IL-2, and IL-4) in peripheral blood mononuclear cells (PBMCs) or splenocytes using quantitative reverse transcription-polymerase chain reaction or enzyme-linked immunospot assays; assessing T-cell proliferation and CD4^+^/CD8^+^ T-cell responses by flow cytometry; and conducting lesion scoring along with histopathological examination of intestinal tissues following challenge to evaluate mucosal protection.

In conclusion, the design and refinement of the MEV targeting the CNA protein demonstrated a strong potential to induce robust immune responses in Th, CTL, and B cells, as indicated by bioinformatics simulations. MEV Construct 8 exhibited stable and high-affinity interactions with immune receptors, reinforcing its viability as a vaccine candidate against *C. perfringens* infections. Additionally, immune simulations suggested that this MEV could effectively elicit a protective immune response in real-world applications, with its antigenicity, safety, and stability validated through assessments of toxicity, allergenicity, solubility, and structural integrity. Although CNA is an essential virulence factor and a promising antigenic target for a mucosal vaccine, broader protection may require the inclusion of additional immunogenic proteins, such as NetB, CPA, FBA, and ZM, in a multi-target vaccine strategy. This work can serve as a modular basis for future vaccine enhancements. While this study provides a comprehensive computational framework, further in vivo studies are necessary to evaluate the vaccine’s safety and efficacy in animal models, particularly in broiler chickens challenged with *C. perfringens*.

## Figures and Tables

**Table 1:** The selected amino acid epitopes specific for T cells and B cells chosen for the construction of the multi-epitope vaccine (MEV).

Number	Epitopes		
	Cytotoxic T Lymphocytes (CTL)	T Helper Lymphocytes (Th)	B Cells
1	EWIAFNPLI	WIAFNPLIAPKLEFT	IGSVDDRYKKESIKPS
2	KMRRVDNTV	SNISVSENKITVNIS	SIPIKDVQFKMRRVDN
3			FKMRRVDNTVIKDGKK

**Table 2:** Physiochemical properties of the multi-epitope vaccine (MEV) constructs estimated by the Expasy-ProtoParam server and Protein-Sol.

Physiochemical Parameters	Values
Molecular weight	12.98 kDa
Isoelectric point	10.26
Extinction coefficient	13,980
Half-life in mammalian reticulocytes, in vitro	1 h
Yeast, in vivo	30 min
*Escherichia coli*, in vivo	>10 h
Instability index	30.28
Aliphatic index	77.91
Grand average of hydropathicity (GRAVY)	−0.617
Solubility	0.848

**Table 3:** Comparison of structural evaluation metrics between refined and unrefined AlphaFold2 protein models.

Parameters	Galaxyrefine Alphafold2 Model (Relaxed Rank2)	Original Alphafold2 Model (Relaxed Rank2)
Residues in core regions	85.40%	75.00%
Residues in allowed regions	13.50%	22.90%
Residues in generously allowed regions	1.00%	1.00%
Residues in disallowed regions	0.00%	1.00%
G-factors (dihedrals, covalent, overall)	−0.09, −0.13, −0.10	−0.58, 0.18, −0.27
Warnings	5	5
Errors	0	2

**Table 4:** The immune simulation parameters used to compare the original collagen adhesin protein (CNA) and four multi-epitope vaccine (MEV) constructs.

Parameters	CNA	Construct 1	Construct 4	Construct 6	Construct 8
IgG + IgM	~75,000	~70,000	~80,000	~76,000	~90,000
B-cell isotype IgM	~325	~375	~375	~360	~350
B-cell isotype IgG1	~50	~50	~60	~60	~100
B-cell isotype IgG2	~0	~0	~0	~0	~0
B-cell (not Memory)	~100	~100	~100	~100	~100
PLB (Plasma) cells	~85	~80	~90	~80	~100
Active B cells	~300	~300	~310	~300	~275
Th memory cells	~1200	~1300	~1400	~1400	~1800
Active CTL cells population	~0.5 (almost 50 percent of total CTL cells are active)	~0.4	~0.4	~0.5	~0.5 (but slightly greater than the rest)

## Data Availability

The original contributions presented in this study are included in the article; further inquiries can be directed to the corresponding authors.

## Supplementary Materials

The following supporting information can be downloaded at: https://www.mdpi.com/article/10.3390/microorganisms13051147/s1. File S1: C-IMMSIM simulation results (1 May 2024); File S2: C-IMMSIM simulation results (29 April 2024); Table S1: Result of BlastP analysis of the genome of *Gallus gallus domesticus* for sequence homology; Table S2: MolProbity, Clash score and GALAXY energy score of the refined models of the selected AlphaFold2 predicted proteins (Relaxed Rank1 and Relaxed Rank 2) obtained from the GalaxyRefine server; Table S3.1: Number of Clusters and Weighted Scores of Different Predicted Docking Models for the MEV and TLR2 Interaction by ClusPro; Table S3.2: Number of Clusters and Weighted Scores of Different Predicted Docking Models for the MEV and TLR5 Interaction by ClusPro; Table S3.3: Number of Clusters and Weighted Scores of Different Predicted Docking Models for the MEV and MHC-I Interaction by ClusPro; Table S3.4: Number of Clusters and Weighted Scores of Different Predicted Docking Models for the MEV and MHC-II Interaction by ClusPro; Figure S1.1: Evaluation of allergenicity of the MEV construct using AllerTOP v2.0 server; Figure S1.2: Evaluation of antigenicity of the MEV construct using VaxiJen server; Figure S2: Secondary Structure of the MEV construct predicted by PSIPRED workbench; Figure S3.1: Molecular Dynamics Simulation Analysis of the Vaccine Construct-TLR2 Complex; Figure S3.2: Molecular Dynamics Simulation Analysis of the Vaccine Construct-MHC-I Complex; Figure S3.3: Molecular Dynamics Simulation Analysis of the Vaccine Construct-MHC-II Complex.

## Abbreviations

Collagen adhesin protein (CNA); cytotoxic T lymphocyte (CTL); fructose-1,6-bisphosphate aldolase (FBA); Food and Drug Administration (FDA); gut-associated lymphoid tissue (GALT); grand average of hydropathicity (GRAVY); helper T lymphocyte (Th); human leukocyte antigen (HLA); interferon (IFN); interleukin (IL); major histocompatibility complex (MHC); molecular dynamics simulation (MDS); multi-epitope vaccine (MEV); National Center for Biotechnology Information (NCBI); necrotic enteritis (NE); necrotic enteritis B-like (NetB); normal mode analysis (NMA); peripheral blood lymphocyte (PBL); protein database (PDB); root mean square deviation (RMSD); toll-like receptor (TLR); zinc metalloprotease (ZM).
