# Supplementary material for: In Silico Design, Optimization, and Evaluation of a Multi-Epitope Vaccine Targeting the Clostridium perfringens Collagen Adhesin Protein
Source: Microorganisms. 2025 May 16;13(5):1147. doi: 10.3390/microorganisms13051147 (PMC12113974; doi:10.3390/microorganisms13051147)
Supplement: Supplementary file 1 [file microorganisms-13-01147-s001.zip › microorganisms-3535256 Supplementary File S1.pdf]

C-IMMSIM simulation results

May 1, 2024

Abstract

This document includes the plots relative to the simulation and the outcome of the epitope/peptide prediction used.

Produced by the C-IMMSIM Online server available at
`http://c-immsim.iac.rm.cnr.it (alias to http://kraken.iac.rm.cnr.it/C-IMMSIM)`

CITATIONS: For publication of results, please cite the following:

Nicolas Rapin, Ole Lund, Massimo Bernaschi, Filippo Castiglione. Computational Immunology Meets Bioinformatics: The Use of Prediction Tools for Molecular Binding in the Simulation of the Immune System. PLoS ONE 5(4): e9862
tt doi:10.1371/journal.pone.0009862, 2010

A retrospective validation

In-silico evaluation of adenoviral COVID-19 vaccination protocols: Assessment of immunological memory up to 6 months after the third dose. P. Stolfi, F. Castiglione, E. Mastrostefano, I. Di Biase, S. Di Biase, G. Palmieri, A. Prisco. Frontiers in Immunology, 13 (2022) doi: 10.3389/fimmu.2022.998262
`https://www.frontiersin.org/articles/10.3389/fimmu.2022.998262i`

An in vivo validation

Identification and validation of viral antigens sharing sequence and structural homology with tumor associated antigens (TAAs). C. Ragone, C. Manolio, B. Cavalluzzo, A. Petrizzo, A. Mauriello, M-L. Tornesello, F. M. Buonaguro, F. Castiglione, L. Vitagliano, M. Ruvo, M. Tagliamonte, L. Buonaguro. Journal for ImmunoTherapy of Cancer. 9:e002694 (2021)
`https://jitc.bmj.com/content/9/5/e002694`

Original C-IMMSIM model: `www.iac.cnr.it/~filippo/c-immsim`

GETTING HELP:
Scientific problems: Filippo Castiglione (filippo dot castiglione at cnr dot it)
Technical problems: Ilaria Gonnella (ilaria dot gonnella at cnr dot it)

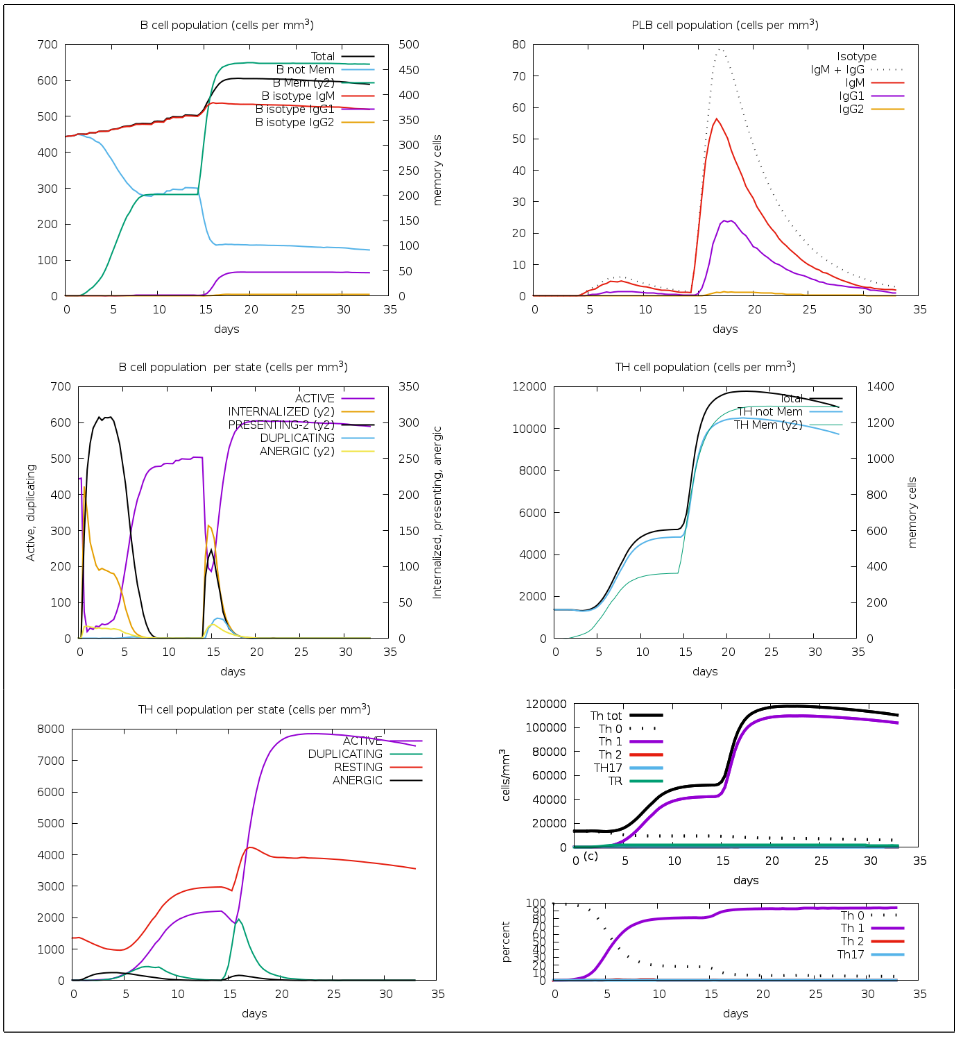

Figure S1.1: Cell counts shown. Legend: Act=active, Intern=internalized the Ag, Pres II = presenting on MHC II, Dup = in the mitotic cycle, Anergic = anergic, Resting = not active.

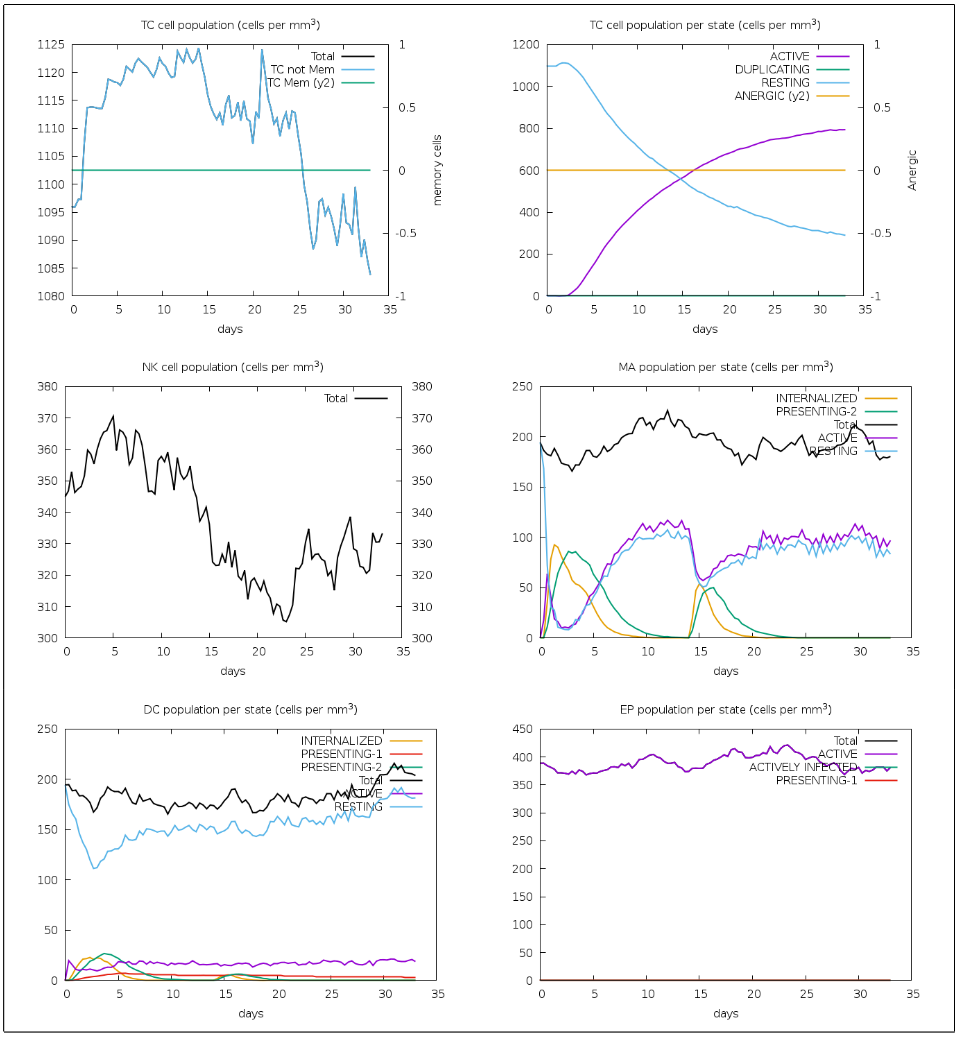

Figure S1.2: Legend: symbols as figure above.

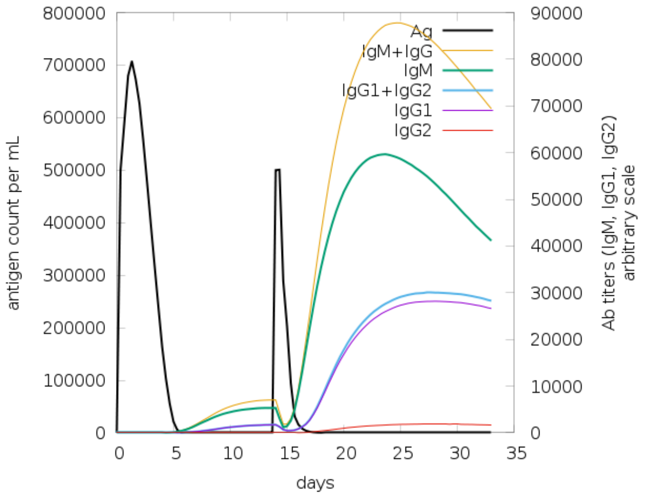

Figure S1.3: The virus, the immunoglobulins and the immunocomplexes.

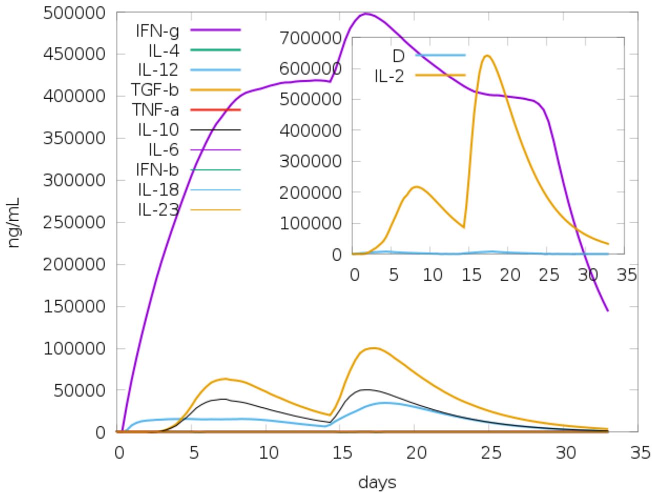

Figure S1.4: Concentration of cytokines and interleukins. Inset plot shows danger signal together with leukocyte growth factor IL-2.

```
Parker_B_scale_epitope_find
Use Parker's propensity scale, takes an antigen block as input,
and creates a list of residues that are possible epitopes.


---------------------------------
Antigen sequence file: /opt/lampp/htdocs/C-IMMSIM/Jobs/input/22061_20240501-171612_5_WzSTJXWFbAe7.FSA_1_001
---------------------------------


EWIAFNPLIAAYKMRRVDNTVGPGPGWIAFNPLIAPKLEFTGPGPGNSNISVSENKITVNIGPGPGSNISVSENKITVNI
SGPGPGENKITVNISQEDSTQKKKVMEKEANENGYLTTYKKIGSVDDRYKKESIKPSKKSIPIKDVQFKMRRVDNKKFKM
RRVDNTVIKDGKK

00000000000000000000100000000000000000000001111100000000000000010100000000000000
00011111000000111111110000001111000000000001011111001110000000000000000001000000
0000001000111
Epitopes of protein 0 ------------
1]       pos=43 len=5       GPGNS
2]       pos=83 len=5       GPGEN
3]       pos=94 len=8       SQEDSTQK
4]       pos=108 len=4       EANE
5]       pos=125 len=5       DDRYK
```

```
DoPeptideList_I:
        Given the antigen injected creates the list of peptides for all the
        NumAgProts proteins and for all i.e., 4 MHCI molecules

        Read class I peptide list from file? NO

=========================================================================

Allele: A0101
Pseudo sequence: KAVHAEQRNKAQTRA
Threshold: 9.456400
Max score: 29.236000


---------------------------------
Antigen sequence file: /opt/lampp/htdocs/C-IMMSIM/Jobs/input/22061_20240501-171612_5_WzSTJXWFbAe7.FSA_1_001
---------------------------------

EWIAFNPLIAAYKMRRVDNTVGPGPGWIAFNPLIAPKLEFTGPGPGNSNISVSENKITVNIGPGPGSNISVSENKITVNI
SGPGPGENKITVNISQEDSTQKKKVMEKEANENGYLTTYKKIGSVDDRYKKESIKPSKKSIPIKDVQFKMRRVDNKKFKM
RRVDNTVIKDGKK

Epitopes of protein 0 ------------
0]       pos=  -1 score=1.000000 unnormalised=104.8410000000        non-binding event

=========================================================================

Allele: A0101
Pseudo sequence: KAVHAEQRNKAQTRA
Threshold: 9.456400
Max score: 29.236000


---------------------------------
```

```
Antigen sequence file: /opt/lampp/htdocs/C-IMMSIM/Jobs/input/22061_20240501-171612_5_WzSTJXWFbAe7.FSA_1_001
---------------------------------

EWIAFNPLIAAYKMRRVDNTVGPGPGWIAFNPLIAPKLEFTGPGPGNSNISVSENKITVNIGPGPGSNISVSENKITVNI
SGPGPGENKITVNISQEDSTQKKKVMEKEANENGYLTTYKKIGSVDDRYKKESIKPSKKSIPIKDVQFKMRRVDNKKFKM
RRVDNTVIKDGKK

Epitopes of protein 0 ------------
0]        pos=  -1 score=1.000000 unnormalised=104.8410000000        non-binding event

========================================================================

Allele: B0702
Pseudo sequence: KAAREEQQIKAQTRE
Threshold: 8.702800
Max score: 28.406000


---------------------------------
Antigen sequence file: /opt/lampp/htdocs/C-IMMSIM/Jobs/input/22061_20240501-171612_5_WzSTJXWFbAe7.FSA_1_001
---------------------------------

EWIAFNPLIAAYKMRRVDNTVGPGPGWIAFNPLIAPKLEFTGPGPGNSNISVSENKITVNIGPGPGSNISVSENKITVNI
SGPGPGENKITVNISQEDSTQKKKVMEKEANENGYLTTYKKIGSVDDRYKKESIKPSKKSIPIKDVQFKMRRVDNKKFKM
RRVDNTVIKDGKK

Epitopes of protein 0 ------------
0]        pos=  21 score=0.013938 unnormalised=1.5402000000        GPGPGWIAF
1]        pos= 134 score=0.037278 unnormalised=4.1192000000        KPSKKSIPI
2]        pos=  -1 score=0.948784 unnormalised=104.8410000000        non-binding event

========================================================================

Allele: B0702
Pseudo sequence: KAAREEQQIKAQTRE
Threshold: 8.702800
Max score: 28.406000


---------------------------------
Antigen sequence file: /opt/lampp/htdocs/C-IMMSIM/Jobs/input/22061_20240501-171612_5_WzSTJXWFbAe7.FSA_1_001
---------------------------------

EWIAFNPLIAAYKMRRVDNTVGPGPGWIAFNPLIAPKLEFTGPGPGNSNISVSENKITVNIGPGPGSNISVSENKITVNI
SGPGPGENKITVNISQEDSTQKKKVMEKEANENGYLTTYKKIGSVDDRYKKESIKPSKKSIPIKDVQFKMRRVDNKKFKM
RRVDNTVIKDGKK

Epitopes of protein 0 ------------
0]        pos=  21 score=0.013938 unnormalised=1.5402000000        GPGPGWIAF
1]        pos= 134 score=0.037278 unnormalised=4.1192000000        KPSKKSIPI
2]        pos=  -1 score=0.948784 unnormalised=104.8410000000        non-binding event
```

```
DoPeptideList_II:
        Given the antigen injected creates the list of peptides for all the
        NumAgProts proteins and for all i.e., 2 MHCII molecules

        Read class II peptide list from file? NO

========================================================================
```

```
Allele: DRB1_0101
Pseudo sequence: KAFAHVEQRKAQTRV
Threshold: 2.392440
Max score: 26.461000


----------------------------------
Antigen sequence file: /opt/lampp/htdocs/C-IMMSIM/Jobs/input/22061_20240501-171612_5_WzSTJXWFbAe7.FSA_1_001
----------------------------------

EWIAFNPLIAAYKMRRVDNTVGPGPGWIAFNPLIAPKLEFTGPGPGNSNISVSENKITVNIGPGPGSNISVSENKITVNI
SGPGPGENKITVNISQEDSTQKKKVMEKEANENGYLTTYKKIGSVDDRYKKESIKPSKKSIPIKDVQFKMRRVDNKKFKM
RRVDNTVIKDGKK

Epitopes of protein 0 ------------
0]        pos=   1 score=0.019920 unnormalised=1.7135600000        WIAFNPLIA
1]        pos=   4 score=0.040845 unnormalised=3.5135600000        FNPLIAAYK
2]        pos=  26 score=0.019920 unnormalised=1.7135600000        WIAFNPLIA
3]        pos=  29 score=0.122965 unnormalised=10.5775600000        FNPLIAPKL
4]        pos=  39 score=0.013317 unnormalised=1.1455600000        FTGPGPGNS
5]        pos=  58 score=0.000030 unnormalised=0.0025600000        VNIGPGPGS
6]        pos= 159 score=0.013794 unnormalised=1.1865600000        MRRVDNTVI
7]        pos=  -1 score=0.769208 unnormalised=66.1680000000        non-binding event

=========================================================================
```


```
Allele: DRB1_0101
Pseudo sequence: KAFAHVEQRKAQTRV
Threshold: 2.392440
Max score: 26.461000


----------------------------------
Antigen sequence file: /opt/lampp/htdocs/C-IMMSIM/Jobs/input/22061_20240501-171612_5_WzSTJXWFbAe7.FSA_1_001
----------------------------------

EWIAFNPLIAAYKMRRVDNTVGPGPGWIAFNPLIAPKLEFTGPGPGNSNISVSENKITVNIGPGPGSNISVSENKITVNI
SGPGPGENKITVNISQEDSTQKKKVMEKEANENGYLTTYKKIGSVDDRYKKESIKPSKKSIPIKDVQFKMRRVDNKKFKM
RRVDNTVIKDGKK

Epitopes of protein 0 ------------
0]        pos=   1 score=0.019920 unnormalised=1.7135600000        WIAFNPLIA
1]        pos=   4 score=0.040845 unnormalised=3.5135600000        FNPLIAAYK
2]        pos=  26 score=0.019920 unnormalised=1.7135600000        WIAFNPLIA
3]        pos=  29 score=0.122965 unnormalised=10.5775600000        FNPLIAPKL
4]        pos=  39 score=0.013317 unnormalised=1.1455600000        FTGPGPGNS
5]        pos=  58 score=0.000030 unnormalised=0.0025600000        VNIGPGPGS
6]        pos= 159 score=0.013794 unnormalised=1.1865600000        MRRVDNTVI
7]        pos=  -1 score=0.769208 unnormalised=66.1680000000        non-binding event
```
