# Supplementary material for: In Silico Design, Optimization, and Evaluation of a Multi-Epitope Vaccine Targeting the Clostridium perfringens Collagen Adhesin Protein
Source: Microorganisms. 2025 May 16;13(5):1147. doi: 10.3390/microorganisms13051147 (PMC12113974; doi:10.3390/microorganisms13051147)
Supplement: Supplementary file 1 [file microorganisms-13-01147-s001.zip › microorganisms-3535256 Supplementary File S2.pdf]

C-IMMSIM simulation results

April 29, 2024

Abstract

This document includes the plots relative to the simulation and the outcome of the epitope/peptide prediction used.

Produced by the C-IMMSIM Online server available at
`http://c-immsim.iac.rm.cnr.it (alias to http://kraken.iac.rm.cnr.it/C-IMMSIM)`

CITATIONS: For publication of results, please cite the following:

Nicolas Rapin, Ole Lund, Massimo Bernaschi, Filippo Castiglione. Computational Immunology Meets Bioinformatics: The Use of Prediction Tools for Molecular Binding in the Simulation of the Immune System. PLoS ONE 5(4): e9862
tt doi:10.1371/journal.pone.0009862, 2010

A retrospective validation

In-silico evaluation of adenoviral COVID-19 vaccination protocols: Assessment of immunological memory up to 6 months after the third dose. P. Stolfi, F. Castiglione, E. Mastrostefano, I. Di Biase, S. Di Biase, G. Palmieri, A. Prisco. Frontiers in Immunology, 13 (2022) doi: 10.3389/fimmu.2022.998262
`https://www.frontiersin.org/articles/10.3389/fimmu.2022.998262i`

An in vivo validation

Identification and validation of viral antigens sharing sequence and structural homology with tumor associated antigens (TAAs). C. Ragone, C. Manolio, B. Cavalluzzo, A. Petrizzo, A. Mauriello, M-L. Tornesello, F. M. Buonaguro, F. Castiglione, L. Vitagliano, M. Ruvo, M. Tagliamonte, L. Buonaguro. Journal for ImmunoTherapy of Cancer. 9:e002694 (2021)
`https://jitc.bmj.com/content/9/5/e002694`

Original C-IMMSIM model: `www.iac.cnr.it/∼filippo/c-immsim`

GETTING HELP:
Scientific problems: Filippo Castiglione (filippo dot castiglione at cnr dot it)
Technical problems: Ilaria Gonnella (ilaria dot gonnella at cnr dot it)

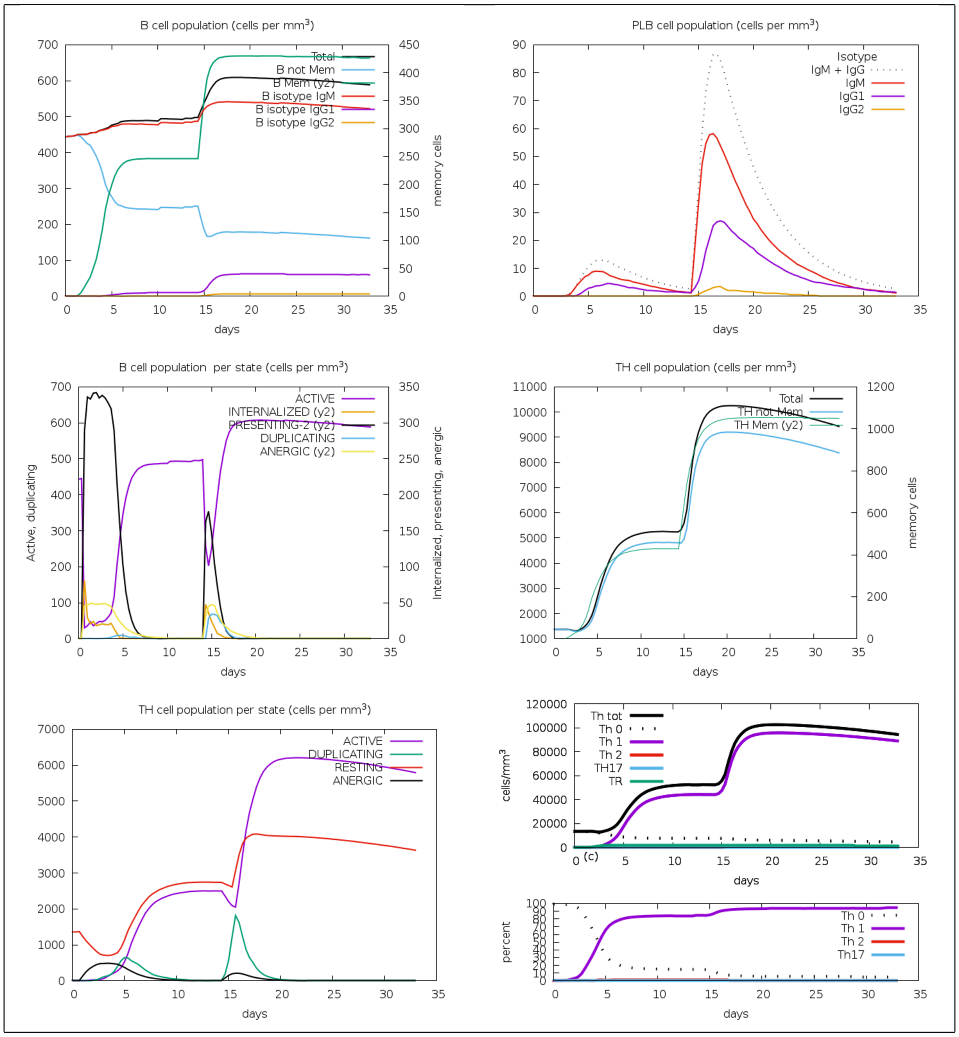

Figure S2.1: Cell counts shown. Legend: Act=active, Intern=internalized the Ag, Pres II = presenting on MHC II, Dup = in the mitotic cycle, Anergic = anergic, Resting = not active.

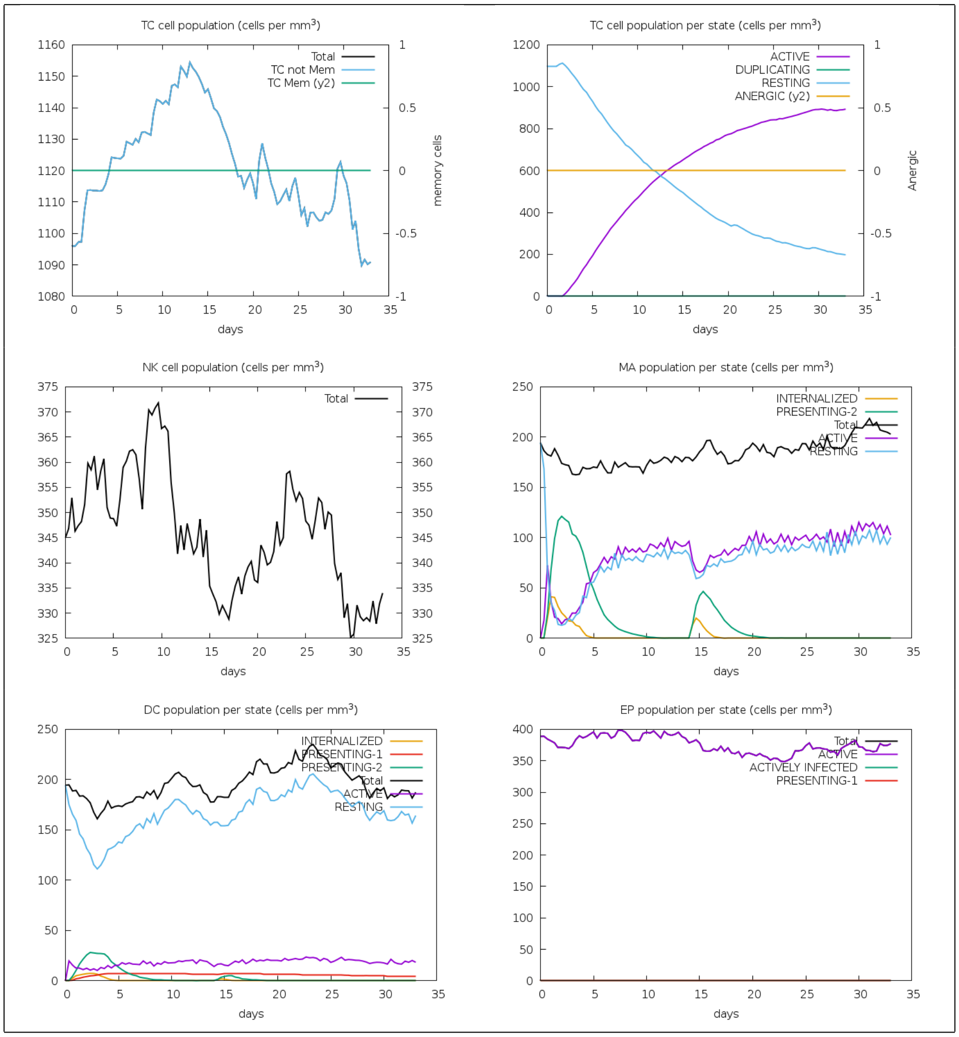

Figure S2.2: Legend: symbols as figure above.

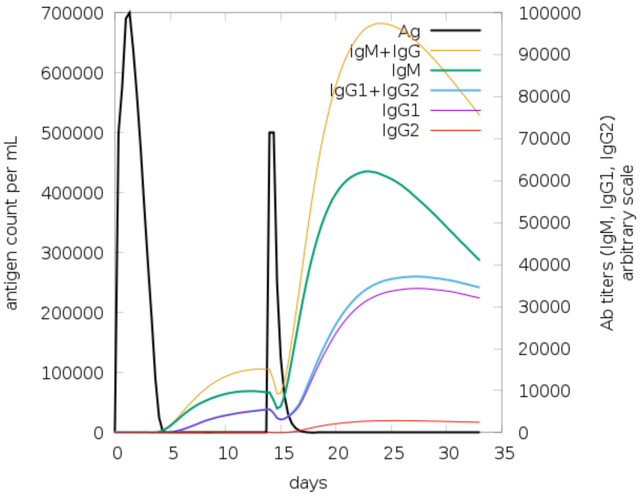

Figure S2.3: The virus, the immunoglobulins and the immunocomplexes.

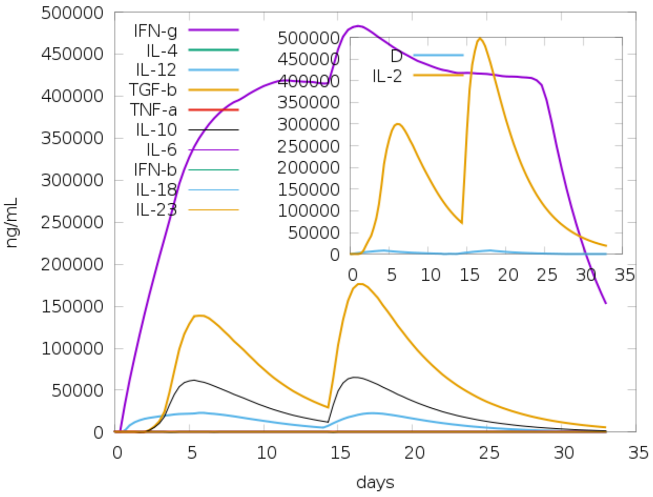

Figure S2.4: Concentration of cytokines and interleukins. Inset plot shows danger signal together with leukocyte growth factor IL-2.

```
Parker_B_scale_epitope_find
Use Parker’s propensity scale, takes an antigen block as input,
and creates a list of residues that are possible epitopes.


----------------------------------
Antigen sequence file: /opt/lampp/htdocs/C-IMMSIM/Jobs/input/21989_20240429-233108_5_Zn6UyXKfE6lp.FSA_1_001
----------------------------------


MSSIQRGRDISNEVVTSLVATPNSINDGGNVQVRLEFKENHQRNIQSGDTITVKWTNSGEVFFEGYEKTIPLYIKDQNVG
QAVIEKTGATLTFNDKIDKLDDVGGWATFTLQGRNITSGNHEHTGIAYIISGSKRADVNITKPESGTTSVFYYKTGSMYT
NDTNHVNWWLLVNPSKVYSEKNVYIQDEIQGGQTLEPDSFEIVVTWYDGYVEKFKGKEAIREFHNKYPNSNISVSENKIT
VNISQEDSTQKFINIFYKTKITNPKQKEFVNNTKAWFKEYNKPAVNGESFNHSVQNINADAGVNGTVKGELKIIKTLKDK
SIPIKDVQFKMRRVDNTVIKDGKKELLLTTDDKGIANVKGLPVGKYEVKEISAPEWIAFNPLIAPKLEFTISDQDTEGKL
WAVENELKTISIPVEKVWVGQTSERAEIKLFADGIEVDKVILNADNNWKHTFENKPEYNSETKQKINYSVSETTISGYES
NITGDAKNGFIVTNTELPDLTIGKEVIGELGDKTKVFNFELTLKQADGKPINGKFNYIGSVDDRYKKESIKPSDGEITFI
EGKATITLSHGQEITIKDLPYGVTYKVMEKEANENGYLTTYNGNNEVTTGELKQDTKVQVVNNKEFVPTTGISTTTEQGT
MVGMVIF

00001101110001000000000111101100000000101100111010000000100000000000000000000110
00000000000000000001000000000000001111111000000000000111000001011110000000000011
11110000000000000011000000000111010110000000000000000000100000000000110000000000
00011111100000000000000001100000000000000100011001100000000000111000100000000100
00000000000000000100011000000001111000000000000010000000000000000000000000111110
00000000000000000000111010000000000100000000010100000101111111110000011001000000
01111110000000010000000000000011000000000000000100100000000000111110011111100000
00000000001000000000000000000000111100000000111111001111011000010000000000111111100
0000000
Epitopes of protein 0 ------------
1]       pos=23 len=4        SIND
2]       pos=114 len=8        NITSGNHE
3]       pos=143 len=4        ESGT
4]       pos=158 len=6        YTNDTN
5]       pos=243 len=6        SQEDST
6]       pos=350 len=4        DDKG
7]       pos=392 len=5        DQDTE
8]       pos=455 len=9        PEYNSETKQ
9]       pos=481 len=6        ITGDAK
10]       pos=541 len=5        DDRYK
11]       pos=548 len=7        SIKPSDG
12]       pos=590 len=4        EANE
13]       pos=601 len=7        NGNNEVT
14]       pos=610 len=4        ELKQ
15]       pos=631 len=7        ISTTTEQ
```

```
DoPeptideList_I:
        Given the antigen injected creates the list of peptides for all the
        NumAgProts proteins and for all i.e., 4 MHCI molecules

        Read class I peptide list from file? NO

=========================================================================

Allele: A0101
Pseudo sequence: KAVHAEQRNKAQTRA
Threshold: 9.456400
Max score: 29.236000
```

```
----------------------------------
Antigen sequence file: /opt/lampp/htdocs/C-IMMSIM/Jobs/input/21989_20240429-233108_5_Zn6UyXKfE6lp.FSA_1_001
----------------------------------

MSSIQRGRDISNEVVTSLVATPNSINDGGNVQVRLEFKENHQRNIQSGDTITVKWTNSGEVFFEGYEKTIPLYIKDQNVG
QAVIEKTGATLTFNDKIDKLDDVGGWATFTLQGRNITSGNHEHTGIAYIISGSKRADVNITKPESGTTSVFYYKTGSMYT
NDTNHVNWWLLVNPSKVYSEKNVYIQDEIQGGQTLEPDSFEIVVTWYDGYVEKFKGKEAIREFHNKYPNSNISVSENKIT
VNISQEDSTQKFINIFYKTKITNPKQKEFVNNTKAWFKEYNKPAVNGESFNHSVQNINADAGVNGTVKGELKIIKTLKDK
SIPIKDVQFKMRRVDNTVIKDGKKELLLTTDDKGIANVKGLPVGKYEVKEISAPEWIAFNPLIAPKLEFTISDQDTEGKL
WAVENELKTISIPVEKVWVGQTSERAEIKLFADGIEVDKVILNADNNWKHTFENKPEYNSETKQKINYSVSETTISGYES
NITGDAKNGFIVTNTELPDLTIGKEVIGELGDKTKVFNFELTLKQADGKPINGKFNYIGSVDDRYKKESIKPSDGEITFI
EGKATITLSHGQEITIKDLPYGVTYKVMEKEANENGYLTTYNGNNEVTTGELKQDTKVQVVNNKEFVPTTGISTTTEQGT
MVGMVIF

Epitopes of protein 0 ------------
0]        pos= 143 score=0.021780 unnormalised=2.4316000000        ESGTTSVFY
1]        pos= 449 score=0.018707 unnormalised=2.0886000000        HTFENKPEY
2]        pos= 469 score=0.019012 unnormalised=2.1226000000        VSETTISGY
3]        pos= 536 score=0.001447 unnormalised=0.1616000000        YIGSVDDRY
4]        pos=  -1 score=0.939053 unnormalised=104.8410000000        non-binding event

========================================================================

Allele: A0101
Pseudo sequence: KAVHAEQRNKAQTRA
Threshold: 9.456400
Max score: 29.236000


----------------------------------
Antigen sequence file: /opt/lampp/htdocs/C-IMMSIM/Jobs/input/21989_20240429-233108_5_Zn6UyXKfE6lp.FSA_1_001
----------------------------------

MSSIQRGRDISNEVVTSLVATPNSINDGGNVQVRLEFKENHQRNIQSGDTITVKWTNSGEVFFEGYEKTIPLYIKDQNVG
QAVIEKTGATLTFNDKIDKLDDVGGWATFTLQGRNITSGNHEHTGIAYIISGSKRADVNITKPESGTTSVFYYKTGSMYT
NDTNHVNWWLLVNPSKVYSEKNVYIQDEIQGGQTLEPDSFEIVVTWYDGYVEKFKGKEAIREFHNKYPNSNISVSENKIT
VNISQEDSTQKFINIFYKTKITNPKQKEFVNNTKAWFKEYNKPAVNGESFNHSVQNINADAGVNGTVKGELKIIKTLKDK
SIPIKDVQFKMRRVDNTVIKDGKKELLLTTDDKGIANVKGLPVGKYEVKEISAPEWIAFNPLIAPKLEFTISDQDTEGKL
WAVENELKTISIPVEKVWVGQTSERAEIKLFADGIEVDKVILNADNNWKHTFENKPEYNSETKQKINYSVSETTISGYES
NITGDAKNGFIVTNTELPDLTIGKEVIGELGDKTKVFNFELTLKQADGKPINGKFNYIGSVDDRYKKESIKPSDGEITFI
EGKATITLSHGQEITIKDLPYGVTYKVMEKEANENGYLTTYNGNNEVTTGELKQDTKVQVVNNKEFVPTTGISTTTEQGT
MVGMVIF

Epitopes of protein 0 ------------
0]        pos= 143 score=0.021780 unnormalised=2.4316000000        ESGTTSVFY
1]        pos= 449 score=0.018707 unnormalised=2.0886000000        HTFENKPEY
2]        pos= 469 score=0.019012 unnormalised=2.1226000000        VSETTISGY
3]        pos= 536 score=0.001447 unnormalised=0.1616000000        YIGSVDDRY
4]        pos=  -1 score=0.939053 unnormalised=104.8410000000        non-binding event

========================================================================

Allele: B0702
Pseudo sequence: KAAREEQQIKAQTRE
Threshold: 8.702800
Max score: 28.406000


----------------------------------
Antigen sequence file: /opt/lampp/htdocs/C-IMMSIM/Jobs/input/21989_20240429-233108_5_Zn6UyXKfE6lp.FSA_1_001
----------------------------------

MSSIQRGRDISNEVVTSLVATPNSINDGGNVQVRLEFKENHQRNIQSGDTITVKWTNSGEVFFEGYEKTIPLYIKDQNVG
```

```
QAVIEKTGATLTFNDKIDKLDDVGGWATFTLQGRNITSGNHEHTGIAYIISGSKRADVNITKPESGTTSVFYYKTGSMYT
NDTNHVNWWLLVNPSKVYSEKNVYIQDEIQGGQTLEPDSFEIVVTWYDGYVEKFKGKEAIREFHNKYPNSNISVSENKIT
VNISQEDSTQKFINIFYKTKITNPKQKEFVNNTKAWFKEYNKPAVNGESFNHSVQNINADAGVNGTVKGELKIIKTLKDK
SIPIKDVQFKMRRVDNTVIKDGKKELLLTTDDKGIANVKGLPVGKYEVKEISAPEWIAFNPLIAPKLEFTISDQDTEGKL
WAVENELKTISIPVEKVWVGQTSERAEIKLFADGIEVDKVILNADNNWKHTFENKPEYNSETKQKINYSVSETTISGYES
NITGDAKNGFIVTNTELPDLTIGKEVIGELGDKTKVFNFELTLKQADGKPINGKFNYIGSVDDRYKKESIKPSDGEITFI
EGKATITLSHGQEITIKDLPYGVTYKVMEKEANENGYLTTYNGNNEVTTGELKQDTKVQVVNNKEFVPTTGISTTTEQGT
MVGMVIF

Epitopes of protein 0 ------------
0]        pos= 141 score=0.043344 unnormalised=4.9192000000        KPESGTTSV
1]        pos= 281 score=0.025317 unnormalised=2.8732000000        KPAVNGESF
2]        pos= 550 score=0.005544 unnormalised=0.6292000000        KPSDGEITF
3]        pos= 578 score=0.002011 unnormalised=0.2282000000        LPYGVTYKV
4]        pos=  -1 score=0.923784 unnormalised=104.8410000000        non-binding event

=========================================================================

Allele: B0702
Pseudo sequence: KAAREEQQIKAQTRE
Threshold: 8.702800
Max score: 28.406000


----------------------------------
Antigen sequence file: /opt/lampp/htdocs/C-IMMSIM/Jobs/input/21989_20240429-233108_5_Zn6UyXKfE6lp.FSA_1_001
----------------------------------

MSSIQRGRDISNEVVTSLVATPNSINDGGNVQVRLEFKENHQRNIQSGDTITVKWTNSGEVFFEGYEKTIPLYIKDQNVG
QAVIEKTGATLTFNDKIDKLDDVGGWATFTLQGRNITSGNHEHTGIAYIISGSKRADVNITKPESGTTSVFYYKTGSMYT
NDTNHVNWWLLVNPSKVYSEKNVYIQDEIQGGQTLEPDSFEIVVTWYDGYVEKFKGKEAIREFHNKYPNSNISVSENKIT
VNISQEDSTQKFINIFYKTKITNPKQKEFVNNTKAWFKEYNKPAVNGESFNHSVQNINADAGVNGTVKGELKIIKTLKDK
SIPIKDVQFKMRRVDNTVIKDGKKELLLTTDDKGIANVKGLPVGKYEVKEISAPEWIAFNPLIAPKLEFTISDQDTEGKL
WAVENELKTISIPVEKVWVGQTSERAEIKLFADGIEVDKVILNADNNWKHTFENKPEYNSETKQKINYSVSETTISGYES
NITGDAKNGFIVTNTELPDLTIGKEVIGELGDKTKVFNFELTLKQADGKPINGKFNYIGSVDDRYKKESIKPSDGEITFI
EGKATITLSHGQEITIKDLPYGVTYKVMEKEANENGYLTTYNGNNEVTTGELKQDTKVQVVNNKEFVPTTGISTTTEQGT
MVGMVIF

Epitopes of protein 0 ------------
0]        pos= 141 score=0.043344 unnormalised=4.9192000000        KPESGTTSV
1]        pos= 281 score=0.025317 unnormalised=2.8732000000        KPAVNGESF
2]        pos= 550 score=0.005544 unnormalised=0.6292000000        KPSDGEITF
3]        pos= 578 score=0.002011 unnormalised=0.2282000000        LPYGVTYKV
4]        pos=  -1 score=0.923784 unnormalised=104.8410000000        non-binding event
```

```
DoPeptideList_II:
        Given the antigen injected creates the list of peptides for all the
        NumAgProts proteins and for all i.e., 2 MHCII molecules

        Read class II peptide list from file? NO

=========================================================================

Allele: DRB1_0101
Pseudo sequence: KAFAHVEQRKAQTRV
Threshold: 2.392440
Max score: 26.461000


----------------------------------
```

```
Antigen sequence file: /opt/lampp/htdocs/C-IMMSIM/Jobs/input/21989_20240429-233108_5_Zn6UyXKfE6lp.FSA_1_001
----------------------------------

MSSIQRGRDISNEVVTSLVATPNSINDGGNVQVRLEFKENHQRNIQSGDTITVKWTNSGEVFFEGYEKTIPLYIKDQNVG
QAVIEKTGATLTFNDKIDKLDDVGGWATFTLQGRNITSGNHEHTGIAYIISGSKRADVNITKPESGTTSVFYYKTGSMYT
NDTNHVNWWLLVNPSKVYSEKNVYIQDEIQGGQTLEPDSFEIVVTWYDGYVEKFKGKEAIREFHNKYPNSNISVSENKIT
VNISQEDSTQKFINIFYKTKITNPKQKEFVNNTKAWFKEYNKPAVNGESFNHSVQNINADAGVNGTVKGELKIIKTLKDK
SIPIKDVQFKMRRVDNTVIKDGKKELLLTTDDKGIANVKGLPVGKYEVKEISAPEWIAFNPLIAPKLEFTISDQDTEGKL
WAVENELKTISIPVEKVWVGQTSERAEIKLFADGIEVDKVILNADNNWKHTFENKPEYNSETKQKINYSVSETTISGYES
NITGDAKNGFIVTNTELPDLTIGKEVIGELGDKTKVFNFELTLKQADGKPINGKFNYIGSVDDRYKKESIKPSDGEITFI
EGKATITLSHGQEITIKDLPYGVTYKVMEKEANENGYLTTYNGNNEVTTGELKQDTKVQVVNNKEFVPTTGISTTTEQGT
MVGMVIF

Epitopes of protein 0 ------------
0]       pos=  14 score=0.016421 unnormalised=2.3335600000       VTSLVATPN
1]       pos=  17 score=0.019067 unnormalised=2.7095600000       LVATPNSIN
2]       pos=  65 score=0.020467 unnormalised=2.9085600000       YEKTIPLYI
3]       pos=  83 score=0.018546 unnormalised=2.6355600000       IEKTGATLT
4]       pos= 127 score=0.042788 unnormalised=6.0805600000       YIISGSKRA
5]       pos= 151 score=0.049149 unnormalised=6.9845600000       YYKTGSMYT
6]       pos= 168 score=0.055736 unnormalised=7.9205600000       WLLVNPSKV
7]       pos= 176 score=0.005472 unnormalised=0.7775600000       VYSEKNVYI
8]       pos= 222 score=0.011200 unnormalised=1.5915600000       FHNKYPNSN
9]       pos= 275 score=0.004564 unnormalised=0.6485600000       WFKEYNKPA
10]       pos= 330 score=0.008350 unnormalised=1.1865600000       MRRVDNTVI
11]       pos= 354 score=0.034414 unnormalised=4.8905600000       IANVKGLPV
12]       pos= 375 score=0.012058 unnormalised=1.7135600000       WIAFNPLIA
13]       pos= 378 score=0.074433 unnormalised=10.5775600000       FNPLIAPKL
14]       pos= 417 score=0.065334 unnormalised=9.2845600000       WVGQTSERA
15]       pos= 465 score=0.014648 unnormalised=2.0815600000       INYSVSETT
16]       pos= 467 score=0.001130 unnormalised=0.1605600000       YSVSETTIS
17]       pos= 522 score=0.017082 unnormalised=2.4275600000       LKQADGKPI
18]       pos= 556 score=0.012459 unnormalised=1.7705600000       ITFIEGKAT
19]       pos= 558 score=0.007808 unnormalised=1.1095600000       FIEGKATIT
20]       pos= 625 score=0.043260 unnormalised=6.1475600000       FVPTTGIST
21]       pos=  -1 score=0.465615 unnormalised=66.1680000000       non-binding event

=========================================================================

Allele: DRB1_0101
Pseudo sequence: KAFAHVEQRKAQTRV
Threshold: 2.392440
Max score: 26.461000


----------------------------------
Antigen sequence file: /opt/lampp/htdocs/C-IMMSIM/Jobs/input/21989_20240429-233108_5_Zn6UyXKfE6lp.FSA_1_001
----------------------------------

MSSIQRGRDISNEVVTSLVATPNSINDGGNVQVRLEFKENHQRNIQSGDTITVKWTNSGEVFFEGYEKTIPLYIKDQNVG
QAVIEKTGATLTFNDKIDKLDDVGGWATFTLQGRNITSGNHEHTGIAYIISGSKRADVNITKPESGTTSVFYYKTGSMYT
NDTNHVNWWLLVNPSKVYSEKNVYIQDEIQGGQTLEPDSFEIVVTWYDGYVEKFKGKEAIREFHNKYPNSNISVSENKIT
VNISQEDSTQKFINIFYKTKITNPKQKEFVNNTKAWFKEYNKPAVNGESFNHSVQNINADAGVNGTVKGELKIIKTLKDK
SIPIKDVQFKMRRVDNTVIKDGKKELLLTTDDKGIANVKGLPVGKYEVKEISAPEWIAFNPLIAPKLEFTISDQDTEGKL
WAVENELKTISIPVEKVWVGQTSERAEIKLFADGIEVDKVILNADNNWKHTFENKPEYNSETKQKINYSVSETTISGYES
NITGDAKNGFIVTNTELPDLTIGKEVIGELGDKTKVFNFELTLKQADGKPINGKFNYIGSVDDRYKKESIKPSDGEITFI
EGKATITLSHGQEITIKDLPYGVTYKVMEKEANENGYLTTYNGNNEVTTGELKQDTKVQVVNNKEFVPTTGISTTTEQGT
MVGMVIF

Epitopes of protein 0 ------------
0]       pos=  14 score=0.016421 unnormalised=2.3335600000       VTSLVATPN
1]       pos=  17 score=0.019067 unnormalised=2.7095600000       LVATPNSIN
2]       pos=  65 score=0.020467 unnormalised=2.9085600000       YEKTIPLYI
3]       pos=  83 score=0.018546 unnormalised=2.6355600000       IEKTGATLT
4]       pos= 127 score=0.042788 unnormalised=6.0805600000       YIISGSKRA
```

```
5]        pos= 151 score=0.049149 unnormalised=6.9845600000        YYKTGSMYT
6]        pos= 168 score=0.055736 unnormalised=7.9205600000        WLLVNPSKV
7]        pos= 176 score=0.005472 unnormalised=0.7775600000        VYSEKNVYI
8]        pos= 222 score=0.011200 unnormalised=1.5915600000        FHNKYPNSN
9]        pos= 275 score=0.004564 unnormalised=0.6485600000        WFKEYNKPA
10]        pos= 330 score=0.008350 unnormalised=1.1865600000        MRRVDNTVI
11]        pos= 354 score=0.034414 unnormalised=4.8905600000        IANVKGLPV
12]        pos= 375 score=0.012058 unnormalised=1.7135600000        WIAFNPLIA
13]        pos= 378 score=0.074433 unnormalised=10.5775600000        FNPLIAPKL
14]        pos= 417 score=0.065334 unnormalised=9.2845600000        WVGQTSERA
15]        pos= 465 score=0.014648 unnormalised=2.0815600000        INYSVSETT
16]        pos= 467 score=0.001130 unnormalised=0.1605600000        YSVSETTIS
17]        pos= 522 score=0.017082 unnormalised=2.4275600000        LKQADGKPI
18]        pos= 556 score=0.012459 unnormalised=1.7705600000        ITFIEGKAT
19]        pos= 558 score=0.007808 unnormalised=1.1095600000        FIEGKATIT
20]        pos= 625 score=0.043260 unnormalised=6.1475600000        FVPTTGIST
21]        pos=  -1 score=0.465615 unnormalised=66.1680000000        non-binding event
```
