# Supplementary material for: In Silico Design, Optimization, and Evaluation of a Multi-Epitope Vaccine Targeting the Clostridium perfringens Collagen Adhesin Protein
Source: Microorganisms. 2025 May 16;13(5):1147. doi: 10.3390/microorganisms13051147 (PMC12113974; doi:10.3390/microorganisms13051147)
Supplement: Supplementary file 1 [file microorganisms-13-01147-s001.zip › microorganisms-3535256-supplementary.pdf]

Supplementary Table and Figures

Supplementary Tables

Table S1. Result of BlastP analysis of the genome of *Gallus gallus domesticus* for sequence homology

**Your search is limited to records that include: *Gallus gallus domesticus* (taxid:9031)**	
RID: 2	7NPMR6T016
Job Title:	Protein Sequence
Program:	BLASTP 2.16.1+
Database:	nr All non-redundant GenBank CDS translations+PDB+SwissProt+PIR+PRF excluding environmental samples from WGS projects
Query ID:	lcl|Query_1379993
Length:	115
**No significant similarity found.**	

Table S2. MolProbity, Clash score and GALAXY energy score of the refined models of the selected AlphaFold2 predicted proteins (Relaxed Rank1 and Relaxed Rank 2) obtained from the GalaxyRefine server.

**AlphaFold Protein Models**	**Model**	**RMSD**	**MolProbity**	**Clash score**	**Poor rotamers**	**Rama favored**	**GALAXY energy**
**Relaxed_Rank1**	Initial	0	2.456	3.2	5.9	77.9	-690.99
	MODEL 1	3.734	1.241	1.4	0	94.7	-2514.31
	MODEL 2	4.148	1.196	0.9	0	93.8	-2495.7
	MODEL 3	3.223	1.241	1.4	0	94.7	-2484.48
	MODEL 4	3.1	1.115	0.5	0	92.9	-2481.27
	MODEL 5	4.334	1.029	0.5	0	94.7	-2480.99
	MODEL 6	4.11	1.403	1.9	0	92.9	-2476.13
	MODEL 7	5.246	1.196	0.9	0	93.8	-2474.83
	MODEL 8	2.409	1.149	0.9	0	94.7	-2472.95
	MODEL 9	2.207	1.288	1.4	1	93.8	-2471.81
	MODEL 10	5.315	1.241	1.4	0	94.7	-2470.59
**Relaxed_Rank2**	Initial	0	2.132	1.1	5.9	79.6	-973
	MODEL 1	2.22	0.972	0.5	0	95.6	-2581.36
	MODEL 2	2.965	0.91	0.9	0	97.3	-2580.71
	MODEL 3	2.048	1.092	0.9	0	95.6	-2566.97
	MODEL 4	2.604	1.317	1.9	0	94.7	-2563.87

MODEL 5	2.269	0.91	0.9	0	97.3	-2556.96
MODEL 6	2.375	0.972	0.5	0	95.6	-2553.8
MODEL 7	2.237	0.972	0.5	1	95.6	-2546.21
MODEL 8	2.427	0.91	0.9	0	97.3	-2543.48
MODEL 9	2.892	1.092	0.9	0	95.6	-2537.49
MODEL 10	1.973	0.784	0.9	0	99.1	-2537.16

Table S3.1. Number of Clusters and Weighted Scores of Different Predicted Docking Models for the MEV and TLR2 Interaction by ClusPro

Cluster	Members	Representative	Weighted Score
**0**	157	Center	-940.6
		Lowest Energy	-1046.4
**1**	82	Center	-982.0
		Lowest Energy	-982.0
**2**	63	Center	-989.6
		Lowest Energy	-1101.9
**3**	60	Center	-909.1
		Lowest Energy	-1005.4
**4**	57	Center	-806.2
		Lowest Energy	-960.2
**5**	45	Center	-883.7
		Lowest Energy	-965.8
**6**	39	Center	-956.8
		Lowest Energy	-956.8
**7**	32	Center	-877.6
		Lowest Energy	-1079.4
**8**	29	Center	-852.8

Cluster	Members	Representative	Weighted Score
		Lowest Energy	-977.0
**9**	28	Center	-870.5
		Lowest Energy	-936.1
**10**	24	Center	-808.7
		Lowest Energy	-1041.1
**11**	24	Center	-824.1
		Lowest Energy	-974.2
**12**	24	Center	-805.1
		Lowest Energy	-954.7
**13**	23	Center	-1006.1
		Lowest Energy	-1006.1
**14**	18	Center	-811.1
		Lowest Energy	-919.1
**15**	17	Center	-803.6
		Lowest Energy	-887.3
**16**	17	Center	-836.4
		Lowest Energy	-919.2
**17**	17	Center	-847.0
		Lowest Energy	-864.1
**18**	15	Center	-897.9
		Lowest Energy	-994.4
**19**	13	Center	-810.7
		Lowest Energy	-972.6
**20**	13	Center	-822.5
		Lowest Energy	-892.8
**21**	13	Center	-973.7
		Lowest Energy	-973.7
**22**	12	Center	-851.1
		Lowest Energy	-907.0
**23**	11	Center	-885.0
		Lowest Energy	-988.2
**24**	10	Center	-878.9
		Lowest Energy	-982.3
**25**	10	Center	-845.5
		Lowest Energy	-845.5

Cluster	Members	Representative	Weighted Score
**26**	9	Center	-844.5
		Lowest Energy	-851.7
**27**	9	Center	-807.3
		Lowest Energy	-871.3
**28**	8	Center	-910.0
		Lowest Energy	-910.0
**29**	7	Center	-834.3
		Lowest Energy	-848.3

Table S3.2. Number of Clusters and Weighted Scores of Different Predicted Docking Models for the MEV and TLR5 Interaction by ClusPro

Cluster	Members	Representative	Weighted Score
**0**	84	Center	-808.7
		Lowest Energy	-998.1
**1**	68	Center	-811.3
		Lowest Energy	-1034.4
**2**	60	Center	-821.6
		Lowest Energy	-893.4
**3**	60	Center	-881.5
		Lowest Energy	-1015.3
**4**	51	Center	-865.8
		Lowest Energy	-917.6
**5**	45	Center	-899.7
		Lowest Energy	-1018.6
**6**	40	Center	-919.5
		Lowest Energy	-944.8
**7**	32	Center	-830.9

Cluster	Members	Representative	Weighted Score
		Lowest Energy	-947.4
**8**	32	Center	-955.8
		Lowest Energy	-967.9
**9**	29	Center	-884.9
		Lowest Energy	-996.2
**10**	25	Center	-793.1
		Lowest Energy	-900.7
**11**	23	Center	-848.6
		Lowest Energy	-878.7
**12**	22	Center	-823.1
		Lowest Energy	-921.8
**13**	19	Center	-800.3
		Lowest Energy	-902.3
**14**	17	Center	-827.1
		Lowest Energy	-910.4
**15**	16	Center	-841.6
		Lowest Energy	-877.2
**16**	15	Center	-889.1
		Lowest Energy	-889.1
**17**	14	Center	-815.0
		Lowest Energy	-847.2
**18**	14	Center	-852.9
		Lowest Energy	-871.3
**19**	13	Center	-824.7
		Lowest Energy	-852.4
**20**	13	Center	-827.7
		Lowest Energy	-911.6
**21**	13	Center	-940.4
		Lowest Energy	-940.4
**22**	13	Center	-877.2
		Lowest Energy	-877.2
**23**	13	Center	-833.7

Cluster	Members	Representative	Weighted Score
		Lowest Energy	-857.0
**24**	12	Center	-836.0
		Lowest Energy	-912.0
**25**	12	Center	-822.6
		Lowest Energy	-894.8
**26**	12	Center	-900.5
		Lowest Energy	-900.5
**27**	12	Center	-896.8
		Lowest Energy	-905.0
**28**	12	Center	-863.6
		Lowest Energy	-886.7
**29**	11	Center	-843.8
		Lowest Energy	-858.5

Table S3.3. Number of Clusters and Weighted Scores of Different Predicted Docking Models for the MEV and MHC-I Interaction by ClusPro

Cluster	Members	Representative	Weighted Score
0	72	Center	-732.2
0	72	Lowest Energy	-790.2
1	59	Center	-671.2
1	59	Lowest Energy	-772.7
2	45	Center	-659.9
2	45	Lowest Energy	-775.4
3	34	Center	-671.6
3	34	Lowest Energy	-684.7
4	33	Center	-759.9
4	33	Lowest Energy	-852.5
5	33	Center	-740.9
5	33	Lowest Energy	-753.1
6	31	Center	-823.6
6	31	Lowest Energy	-852.7
7	29	Center	-644
7	29	Lowest Energy	-785.7

8	29	Center	-643.4
8	29	Lowest Energy	-778.8
9	29	Center	-627.7
9	29	Lowest Energy	-836.4
10	29	Center	-890.3
10	29	Lowest Energy	-890.3
11	27	Center	-652.9
11	27	Lowest Energy	-713.8
12	26	Center	-848.5
12	26	Lowest Energy	-872.4
13	20	Center	-636.3
13	20	Lowest Energy	-759.5
14	20	Center	-645.3
14	20	Lowest Energy	-689.9
15	19	Center	-658.6
15	19	Lowest Energy	-745.9
16	19	Center	-741
16	19	Lowest Energy	-741
17	17	Center	-698
17	17	Lowest Energy	-765.4
18	17	Center	-733
18	17	Lowest Energy	-733
19	16	Center	-621.3
19	16	Lowest Energy	-728.6
20	16	Center	-631.2
20	16	Lowest Energy	-749.3
21	14	Center	-748
21	14	Lowest Energy	-748
22	14	Center	-647.7
22	14	Lowest Energy	-698.1
23	13	Center	-647
23	13	Lowest Energy	-686.4
24	11	Center	-667.1
24	11	Lowest Energy	-717.3
25	11	Center	-622.5
25	11	Lowest Energy	-652.9

26	11	Center	-652.6
26	11	Lowest Energy	-674.3
27	10	Center	-648.3
27	10	Lowest Energy	-677
28	10	Center	-722.6
28	10	Lowest Energy	-751.4
29	10	Center	-688.6
29	10	Lowest Energy	-688.6

Table 3.4. Number of Clusters and Weighted Scores of Different Predicted Docking Models for the MEV and MHC-II Interaction by ClusPro

Cluster	Members	Representative	Weighted Score
0	115	Center	-757.1
0	115	Lowest Energy	-794.2
1	98	Center	-660.3
1	98	Lowest Energy	-757.9
2	80	Center	-634.5
2	80	Lowest Energy	-784.7
3	67	Center	-671.4
3	67	Lowest Energy	-708
4	53	Center	-683.6
4	53	Lowest Energy	-832.3
5	47	Center	-629
5	47	Lowest Energy	-751.7
6	44	Center	-659.2
6	44	Lowest Energy	-738.6
7	36	Center	-718.7
7	36	Lowest Energy	-728.7
8	30	Center	-792.7
8	30	Lowest Energy	-792.7
9	28	Center	-633.2
9	28	Lowest Energy	-693.1

10	28	Center	-700
10	28	Lowest Energy	-704.4
11	27	Center	-765.1
11	27	Lowest Energy	-765.1
12	26	Center	-624.8
12	26	Lowest Energy	-704.6
13	23	Center	-692
13	23	Lowest Energy	-692
14	20	Center	-724.9
14	20	Lowest Energy	-724.9
15	20	Center	-712.2
15	20	Lowest Energy	-712.2
16	20	Center	-621.3
16	20	Lowest Energy	-790.7
17	16	Center	-641.6
17	16	Lowest Energy	-747.3
18	13	Center	-690.7
18	13	Lowest Energy	-690.7
19	13	Center	-664.2
19	13	Lowest Energy	-664.2
20	11	Center	-670.4
20	11	Lowest Energy	-726.7
21	11	Center	-628.4
21	11	Lowest Energy	-699.2
22	11	Center	-722
22	11	Lowest Energy	-722
23	11	Center	-708
23	11	Lowest Energy	-708
24	10	Center	-731.5
24	10	Lowest Energy	-731.5
25	9	Center	-679.1
25	9	Lowest Energy	-721.7
26	5	Center	-658.3
26	5	Lowest Energy	-659.8
27	4	Center	-622.7
27	4	Lowest Energy	-688.6

Supplementary Figures

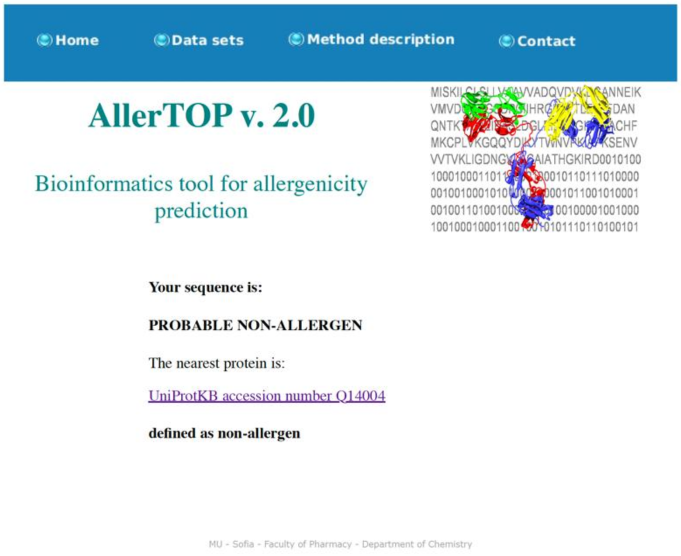

Figure S1.1. Evaluation of allergenicity of the MEV construct using AllerTOP v2.0 server.

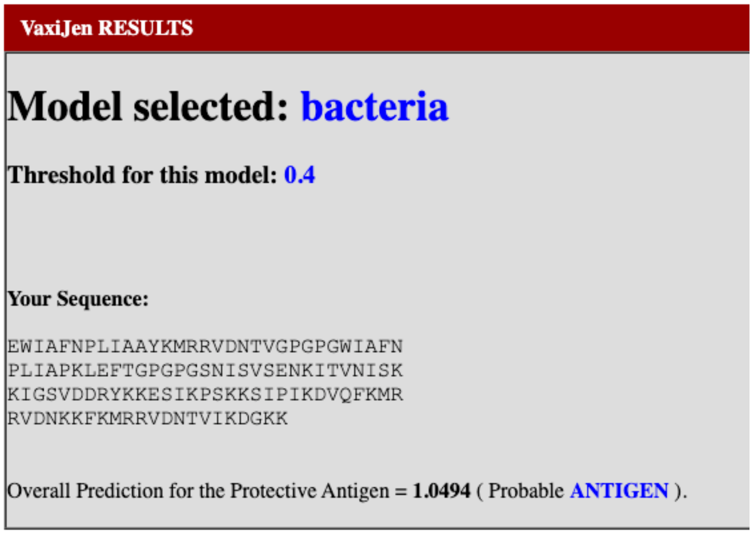

Figure S1.2. Evaluation of antigenicity of the MEV construct using VaxiJen server.

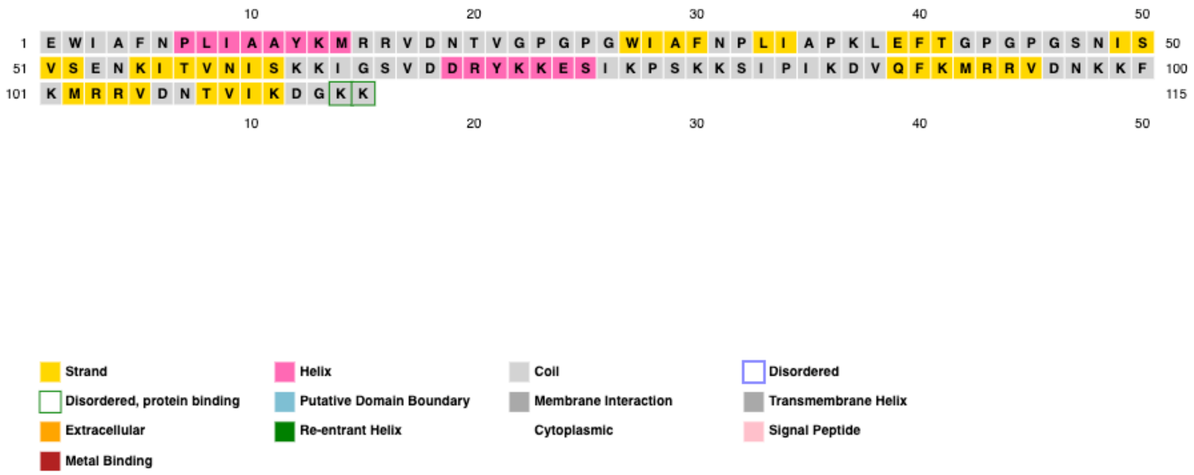

Figure S2. Secondary Structure of the MEV construct predicted by PSIPRED workbench.

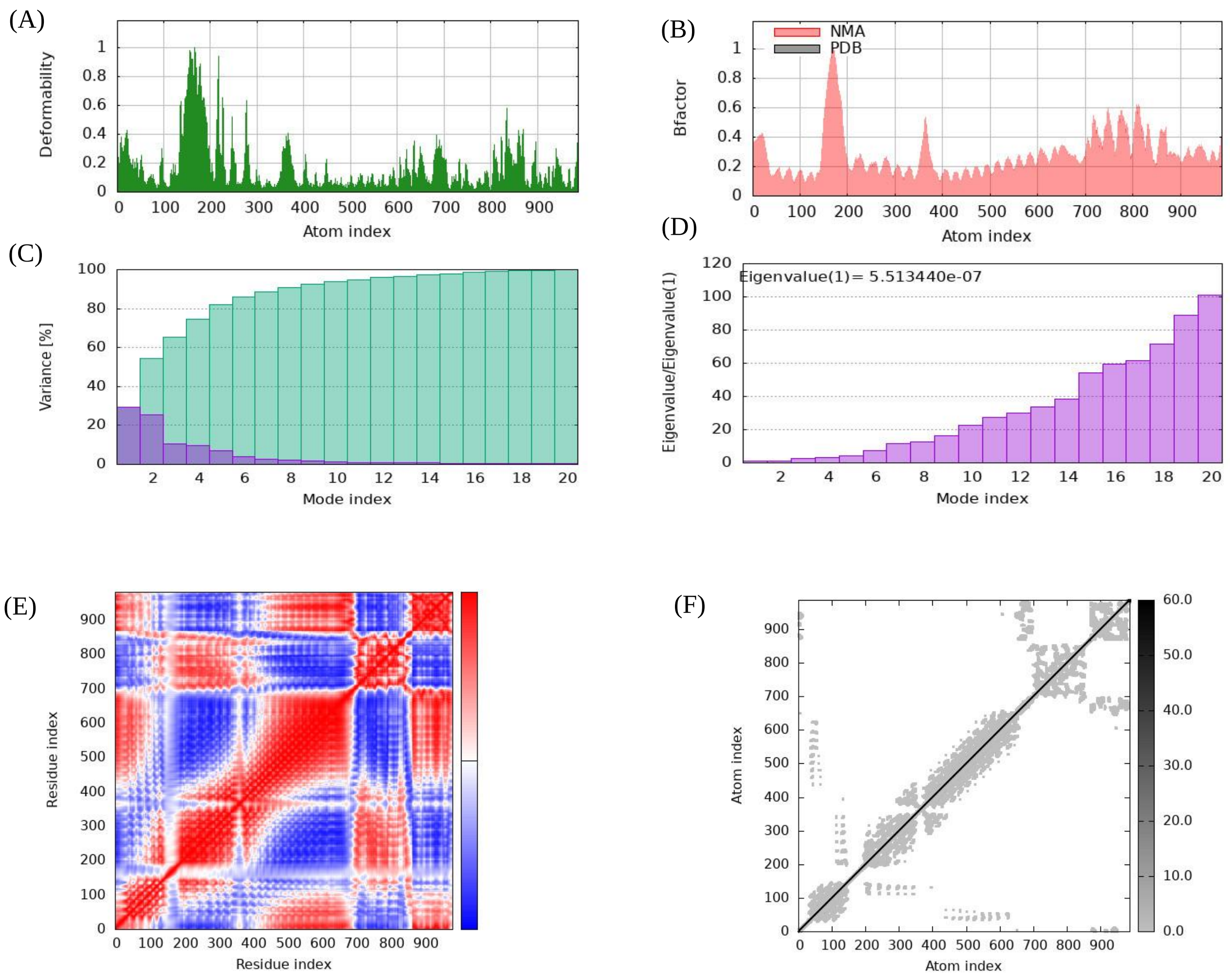

Figure S3.1. Molecular Dynamics Simulation Analysis of the Vaccine Construct-TLR2 Complex. (A) The deformability analysis of the complex, depicting regions of high flexibility. (B) B-factor map showing fluctuations in atomic positions during the simulation. (C) Variance analysis demonstrating the contribution of different modes to the overall motion. (D) Eigenvalue plot illustrating the stiffness and stability of the system based on the principal modes of motion. (E) Covariance matrix illustrating correlated, anti-correlated, and irrelevant motions within the complex in red, blue, and white colors. (F) Elastic network model representing the connections and constraints among residues.

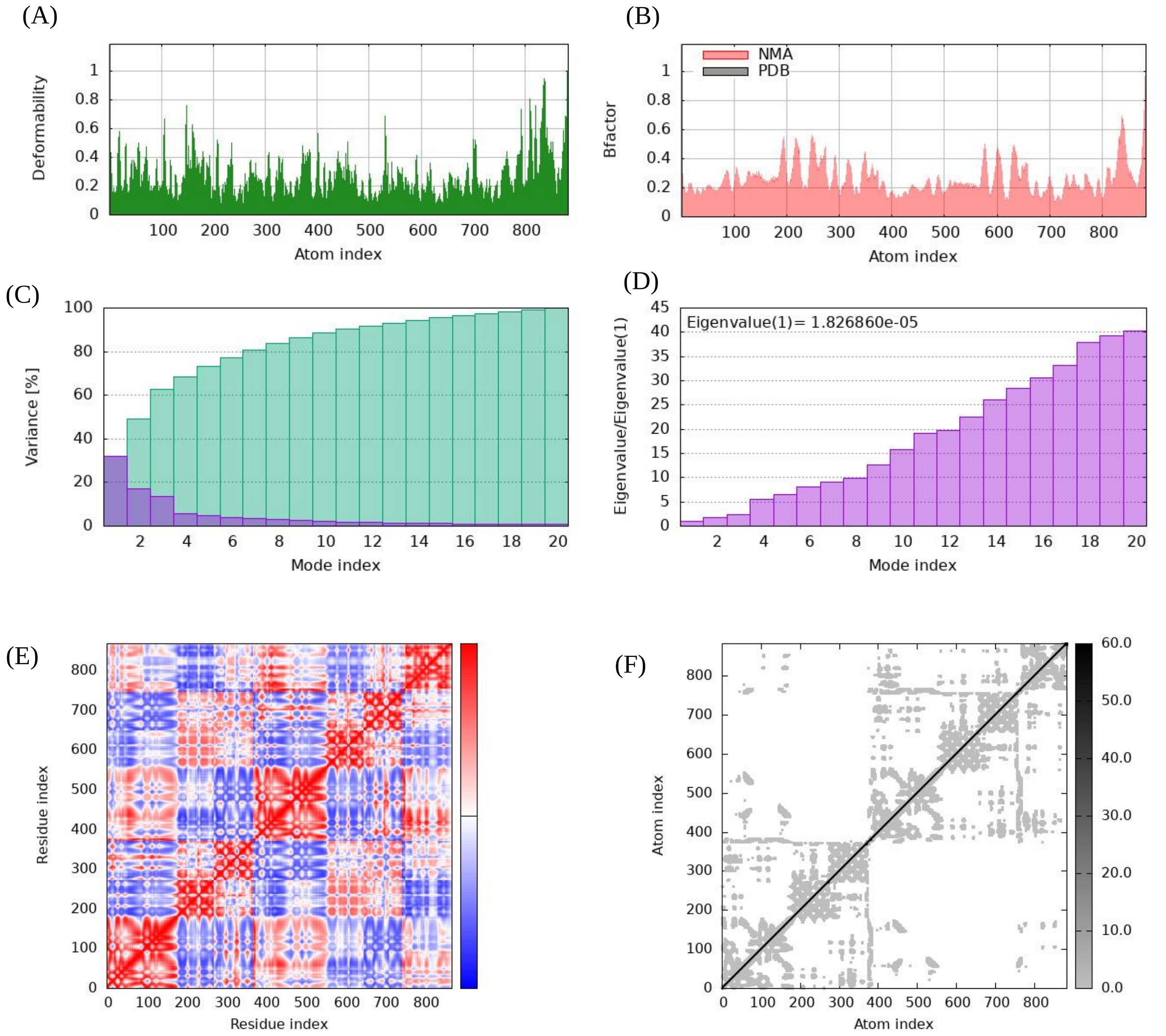

Figure S3.2. Molecular Dynamics Simulation Analysis of the Vaccine Construct-MHC-I Complex. (A) The deformability analysis of the complex, depicting regions of high flexibility. (B) B-factor map showing fluctuations in atomic positions during the simulation. (C) Variance analysis demonstrating the contribution of different modes to the overall motion. (D) Eigenvalue plot illustrating the stiffness and stability of the system based on the principal modes of motion. (E) Covariance matrix illustrating correlated, anti-correlated, and irrelevant motions within the complex in red, blue, and white colors. (F) Elastic network model representing the connections and constraints among residues.

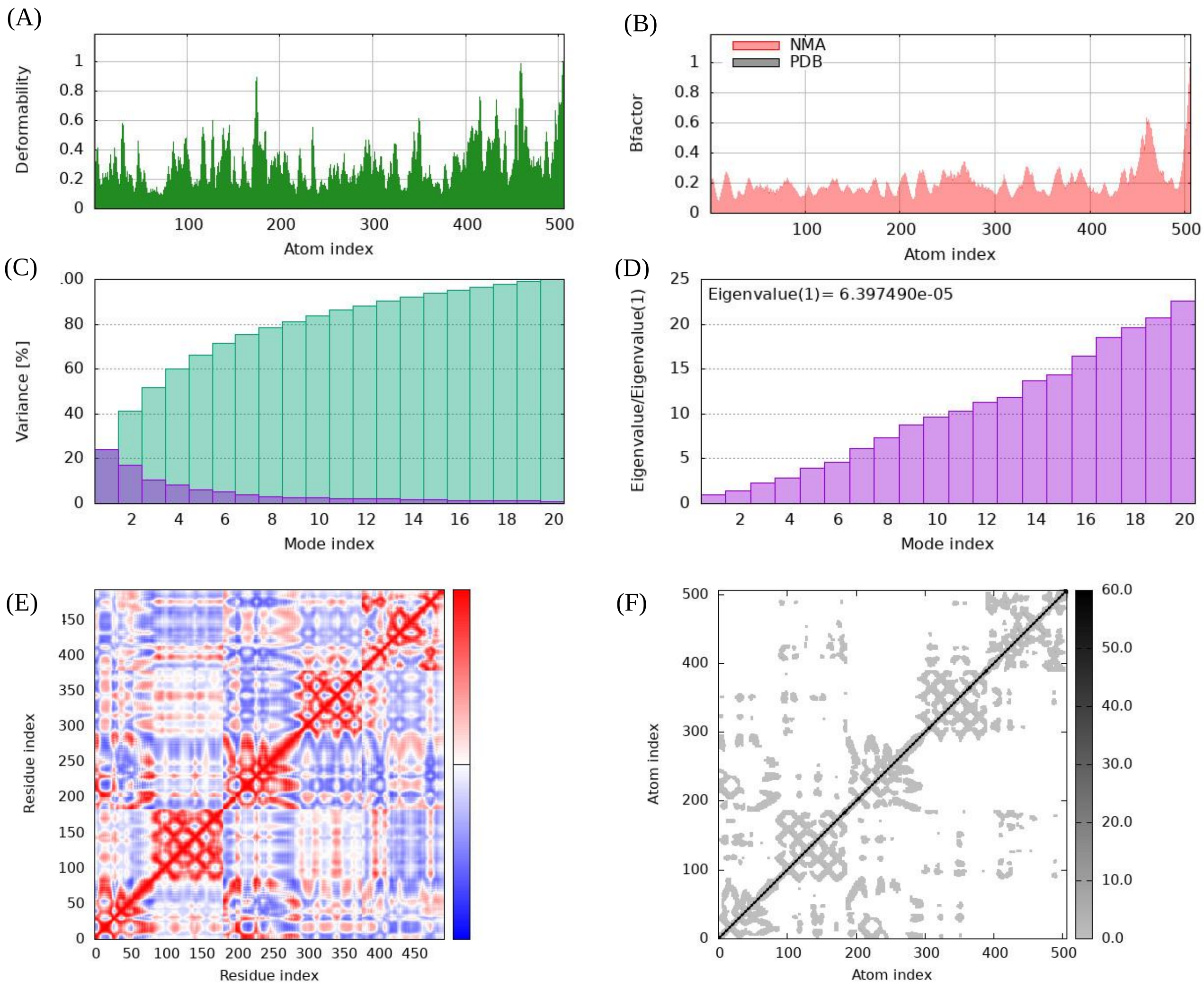

Figure S3.3. Molecular Dynamics Simulation Analysis of the Vaccine Construct-MHC-II Complex. (A) The deformability analysis of the complex, depicting regions of high flexibility. (B) B-factor map showing fluctuations in atomic positions during the simulation. (C) Variance analysis demonstrating the contribution of different modes to the overall motion. (D) Eigenvalue plot illustrating the stiffness and stability of the system based on the principal modes of motion. (E) Covariance matrix illustrating correlated, anti-correlated, and irrelevant motions within the complex in red, blue, and white colors. (F) Elastic network model representing the connections and constraints among residues.
